# Role of cellulose response transporter-like protein CRT2 in cellulase induction in *Trichoderma reesei*

**DOI:** 10.1186/s13068-023-02371-7

**Published:** 2023-07-24

**Authors:** Su Yan, Yan Xu, Xiao-Wei Yu

**Affiliations:** Lab of Brewing Microbiology and Applied Enzymology, School of Biotechnology and Key Laboratory of Industrial Biotechnology of Ministry of Education, Jiangnan University, Wuxi, 214122 China

**Keywords:** *Trichoderma reesei*, Sugar transporter, CRT2, Cellulase induction

## Abstract

**Background:**

Induction of cellulase in cellulolytic fungi *Trichoderma reesei* is strongly activated by cellulosic carbon sources. The transport of cellulosic inducer and the perception of inducing signal is generally considered as the critical process for cellulase induction, that the inducing signal would be perceived by a sugar transporter/transceptor in *T. reesei*. Several sugar transporters are coexpressed during the induction stage, but which function they serve and how they work collaboratively are still difficult to elucidate.

**Results:**

In this study, we found that the constitutive expression of the cellulose response transporter-like protein CRT2 (previously identified as putative lactose permease TRE77517) improves cellulase induction on a cellulose, cellobiose or lactose medium. Functional studies indicate that the membrane-bound CRT2 is not a transporter of cellobiose, lactose or glucose in a yeast system, and it also does not affect cellobiose and lactose utilization in *T. reesei*. Further study reveals that CRT2 has a slightly similar function to the cellobiose transporter CRT1 in cellulase induction. Overexpression of CRT2 led to upregulation of CRT1 and the key transcription factor XYR1. Moreover, overexpression of CRT2 could partially compensate for the function loss of CRT1 on cellulase induction.

**Conclusions:**

Our study uncovers the novel function of CRT2 in cellulase induction collaborated with CRT1 and XYR1, possibly as a signal transductor. These results deepen the understanding of the influence of sugar transporters in cellulase production.

**Supplementary Information:**

The online version contains supplementary material available at 10.1186/s13068-023-02371-7.

## Introduction

Lignocellulose is the most abundant renewable resource in the world. The bioconversion of lignocellulose with cellulases is cost-effective and environmentally friendly, which meets the demand for sustainable development [1]. Over the past decades, the filamentous fungus *Trichoderma reesei* has attracted widespread attention due to its outstanding performance in cellulase production. Cellulase genes in *T. reesei* are highly induced when the mycelia are exposed to cellulose and several soluble disaccharides, but the cellulase genes are repressed by glucose through carbon catabolite repression (CCR) [2]. Several studies have been conducted concerning how *T. reesei* senses insoluble lignocellulose and activates the transcription of downstream cellulase genes. It is hypothesized that released soluble disaccharides from lignocellulose would trigger downstream cellulase induction after their internalization by sugar transporters [3, 4].

Most sugar transporters in eukaryotes belong to the major facilitator superfamily and consist of 12 transmembrane α-helices [3]. These transporters function as porters on the cell surface, gating the cross-membrane transport of sugar and other molecules. Meanwhile, some membrane-bound transporters play important roles in signal sensing and transduction, which are indispensable for the perception of environmental conditions [5]. More than twenty hexose transporters have been identified in *Saccharomyces cerevisiae* and are coordinately regulated. Among those, SNF3 and RGT2 act as sensors of extracellular glucose concentration [6]. In *Neurospora crassa*, the perception of glucose is conducted via the RGT2 homolog RCO3, and the signal could activate the transcription factor COL26 and then regulate the expression of glucose transporter HGT1/2 and GLT1, which forms a dual-affinity glucose transporter system [7, 8]. Moreover, the cellodextrin transporters CDT-1 and CDT-2 in *N. crassa* could efficiently transport cellobiose [9]; although the mutation in CDT-1 or CDT-2 abolishes their cellobiose transport ability, they could still activate the transcription of cellulase genes at levels indistinguishable from the levels of the wild type [5]. These results strongly indicate that the cellodextrin transporters CDT-1 and CDT-2 also act as transceptors for cellulase induction. In addition, the transporter-like membrane protein CLP1 in *N. crassa* could negatively regulate cellulase production with the transporters CDT-1 and CDT-2, although CLP1 cannot transport cellobiose [10].

In *T. reesei*, over 50 sugar transporters have been annotated, but only a few of them have been investigated [11]. The glucose transporter HXT1 is induced at lower glucose concentrations and is regulated by oxygen conditions, which shares a similar expression pattern to the mammalian GLUT1 [12]. Two putative lactose permeases, TRE77517 and TRE79202, were identified due to defects in lactose uptake and cellulase production in knockout mutants [13]. The sugar transporter STP1 is capable of transporting both cellobiose and glucose, its deletion affects the glucose transport and improves cellulase induction on Avicel [14]. Importantly, the cellobiose transporter CRT1 in *T. reesei* is critical for cellulase induction by activating the downstream transcription factor XYR1, and the deletion of *crt1* impairs its cellulase production [14, 15]. A recent study indicated that the function of lactose transport and signal transduction in CRT1 is independent. A truncated form of CRT1 in the C-terminus cannot induce cellulase production, whereas its lactose transport is not affected [16]. These results further confirm the transceptor role of CRT1 in cellulase production, which is similar to the role of CDT-1 and CDT-2 in *N. crassa*. Recently, a transport assay for 18 sugar transporters of *T. reesei* was conducted in *Xenopus laevis* oocytes. These transporters have different preferences for sugars and different transport kinetics [17], providing a solid foundation for the thorough investigation of sugar transporters in *T. reesei*. In addition, several sugar transporters are co-induced in cellulase induction and are also under the regulation of XYR1 [17–19], indicating that these sugar transporters might work in a similar manner in cellulase induction. However, how these transporters regulate cellulase induction collaboratively and which roles they work still need experimental exploration, which is critical for the thorough understanding of the cellulase induction networks in *T. reesei*.

In our previous study, deletion of *vps13* or *vps21* improved cellulase production, which might be attributed to the differentially expressed sugar transporters (Additional file 1: Figure S1) [20]. In this study, the effect of these sugar transporters on cellulase production is investigated, and we found that the constitutive expression of a previously annotated putative lactose permease TRE77517 increases cellulase induction. We designated TRE77517 cellulose response transporter-like protein CRT2. Then, we characterized the sugar transport capacity of CRT2 in *S. cerevisiae* and *T. reesei*, and deciphered its role in cellulase induction with main cellobiose transporter CRT1 and transcription factor XYR1.

## Results

### The effect of sugar transporter dysfunction on cellulase production

**Fig. 1:**
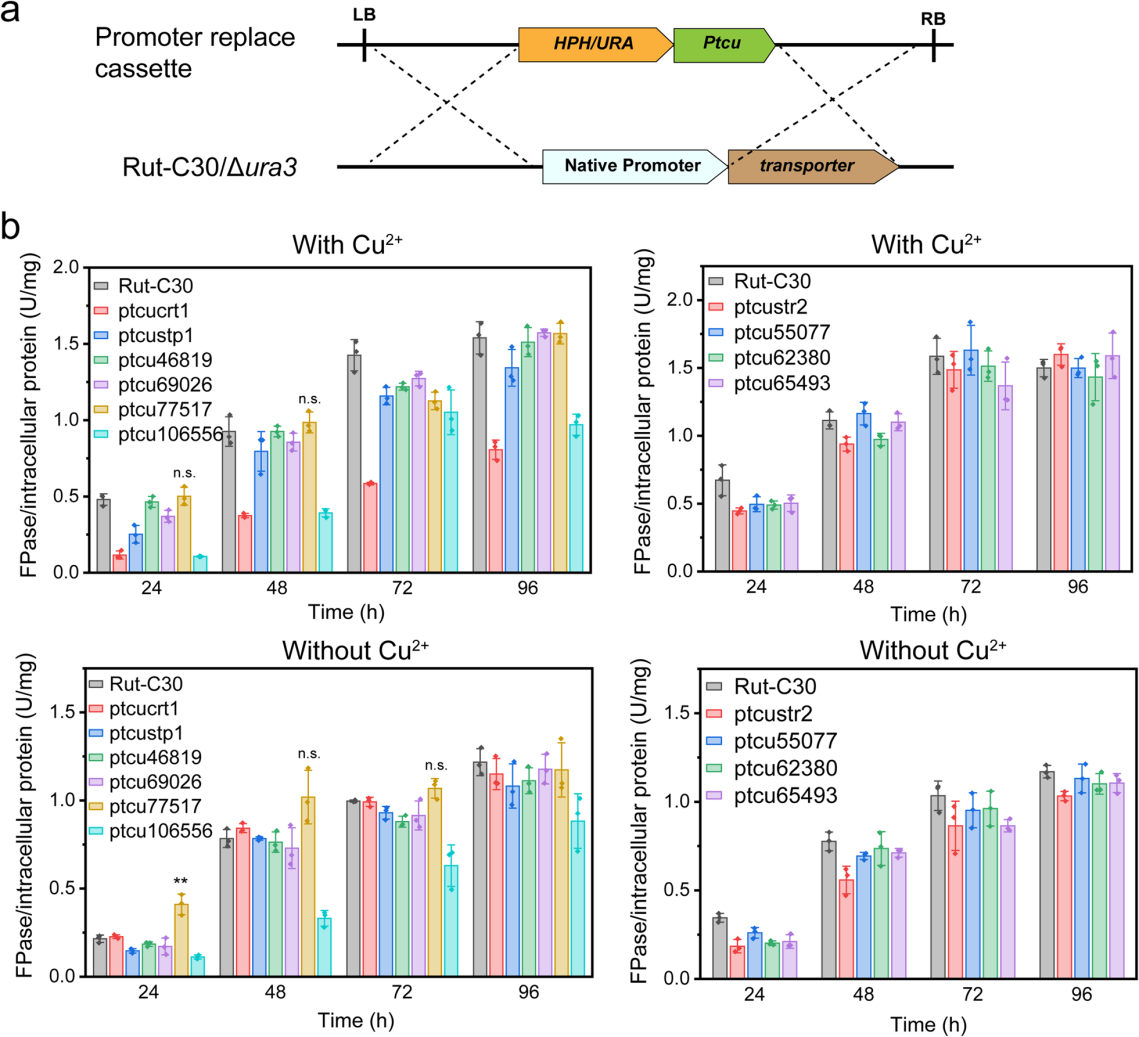
Evaluating the effect on the dysfunction of sugar transporters for cellulase production. **a** Diagram of the promoter replace cassette. The native promoter of each sugar transporter was replaced by the copper-controlled Ptcu promoter. **b** Analysis of cellulase production of Rut-C30, ptcucrt1, ptcustp1, ptcu46819, ptcu69026, ptcu77517, ptcu106556, ptcustr2, ptcu55077, ptcu62380, ptcu65493 in Avicel inducing medium. 20 μM CuSO_4_ was added to repress the expression of the desired sugar transporters. All the transformants and parent strain were precultured in SDB for mycelia accumulation and transferred to Avicel inducing medium for 96 h. The filter paper activity (FPase) was used to represent the cellulase production and normalized to the intracellular protein content, which referred to the mycelia accumulation on Avicel medium. Values are presented as the mean with the standard deviation from three biological replicates (three flasks). Statistical significance was calculated through Student’s *t* test. *p* ≥ 0.5 was indicated as n.s. ** represents *p* < 0.01

In our previous study, the expression of the sugar transporter genes *tre65493*, *tre77517*, *tre69026*, *str2*, *tre106556*, and *tre46819* was upregulated in the single mutant Δ*vps13* or Δ*vps21.* In addition, *tre62380*, *tre55077* and *hxt1* were downregulated in the same period, which probably resulted in increased cellulase production (Additional file 1: Figure S1) [20]. To further understand the function of these sugar transporters in cellulase production, we used a copper-repressed promoter Ptcu to endogenously express these sugar transporters in the parent Rut-C30 (Fig. 1a) [21]. The strains with Ptcu expressed CRT1 or STP1, whose functions have been characterized, were used as controls. The constitutive expression or repression of these sugar transporters were verified by RT-qPCR (Additional file 1: Figure S2). When copper was added in Avicel inducing medium, cellulase production was significantly repressed in the strains ptcucrt1 and ptcu106556, consistent with a previous study showing that cellulase production was impeded when *crt1* was disrupted [14]. When copper was not involved, we found a rapid increase in cellulase production in the strain ptcu77517 only in the initial stage after being transferred to the Avicel-inducing medium, which is a promising candidate for further investigation (Fig. 1b).

### Membrane-bound protein TRE77517 cannot transport mono-/disaccharides

**Fig. 2:**
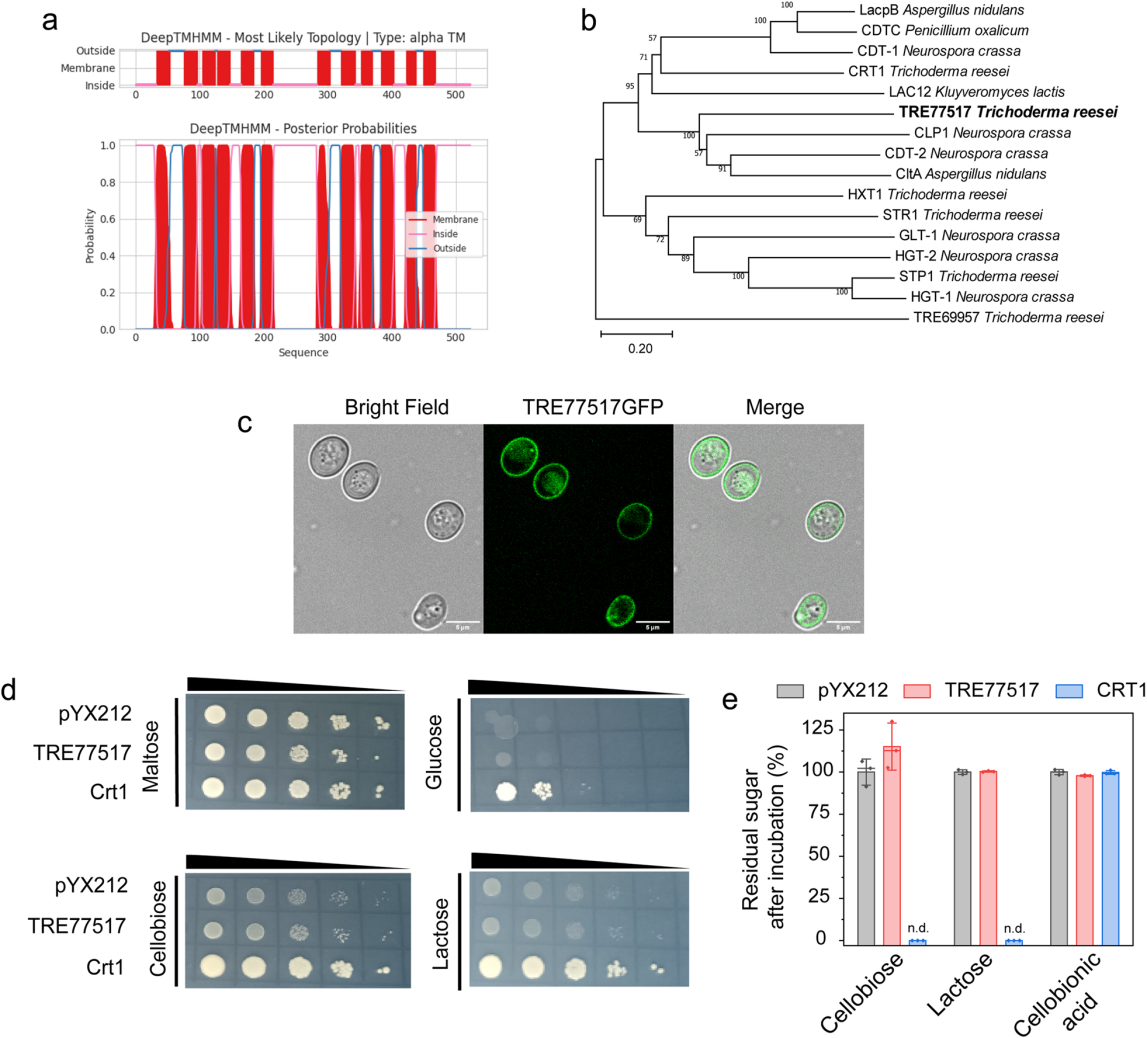
Analysis of sugar transport of TRE77517 in *S. cerevisiae*. **a** Prediction of the transmembrane region of TRE77517 through DeepTMHMM version 1.0.13 (https://dtu.biolib.com/DeepTMHMM). **b** Phylogenetic analysis of TRE77517 and selected sugar transporters. Alignments were conducted by ClustalW, and a phylogenetic tree was generated via MEGA-X software (Version 10.2.5) using the neighbor-joining method with 1000 bootstrap replications. **c** Subcellular location of TRE77517GFP in *S. cerevisiae*. Yeast strains expressing GFP-tagged TRE77517 under strong constitutive promoter Ptpi were cultured in SC medium with 2% maltose as carbon source for 16 h before microscopy analysis. Images was captured with a 63 × oil objective, and the scale bar represents 5 μm. **d** Analysis of sugar transport in *S. cerevisiae*. The yeast EBY.VW4000 coexpressing the β-glucosidases *gh1-1* and TRE77517 were precultured on SC medium with maltose as the carbon source. Cells were harvested and washed twice, tenfold serial dilution was applied, and 5 μL suspension was dropped on the SC plate with maltose, glucose, cellobiose or lactose as the carbon source. The strains on maltose plate were incubated at 30 °C for 3 days. 5 days were applied for strains on glucose, cellobiose and lactose SC plate. The empty plasmid pYX212 was used as a negative control, and a strain harboring the cellobiose transporter CRT1 was used as a positive control. **e** Equal amounts of yeast strains (OD_600_ = 30) were mixed with 200 μM sugar and incubated at 30 °C at 200 rpm for 40 min. The residual sugar in yeast with empty plasmid was normalized as 100%. Residual sugar was analyzed by HPAEC. n.d. for not detected

TRE77517 contains 522 amino acids, which form 12 transmembrane regions predicted by DeepTMHMM version 1.0.13 (https://dtu.biolib.com/DeepTMHMM) (Fig. 2a). TRE77517 was first identified as a putative lactose permease, because lactose consumption and cellulase production on lactose were decreased when *tre77517* was disrupted [13]. A phylogenetic analysis with other characterized lactose/cellobiose transporters indicated that TRE77517 is very close to the cellodextrin transporter CDT-2 in *N. crassa* [5] (Fig. 2b), as well as the cellobiose transporter CltA in *Aspergillus nidulans* [22]. TRE77517 also shows similarity to those lactose transporter LacpB in *A. nidulans* and CRT1 in *T. reesei* [23] (Fig. 2b), indicating that TRE77517 might transport lactose and cellobiose.

The ability of TRE77517 to transport sugar was tested in EBY/gh1-1, which is the hexose transport null *S. cerevisiae* EBY.VW4000 transformed with a β-glucosidase *gh1-1* (NCU00130) from *N. crassa*. The expression of β-glucosidase permits *S. cerevisiae* growth on some disaccharides when disaccharide transporter was co-expressed [9]. Meanwhile, a GFP fused TRE77517 was used to visualize the subcellular distribution in *S. cerevisiae*, and the results indicated the cortical localization of TRE77517GFP, which is consistent with its transmembrane prediction as a membrane protein (Fig. 2c). The *S. cerevisiae* transformant harboring TRE77517 could not grow either on glucose, cellobiose or lactose plates after 5 day culturation compared to the transformant with the empty plasmid pYX212 (Fig. 2d). The sugar uptake assay also indicated that TRE77517 could not transport cellobiose and lactose, which is consistent with the plate growth (Fig. 2e). However, the strain co-expressed with cellobiose transporter CRT1 could successfully transport glucose, cellobiose and lactose [15, 17] (Fig. 2d, e). Other sugars were also tested, but the result indicated that TRE77517 cannot support growth on galactose, mannose, fructose, or sucrose (Additional file 1: Figure S3). Meanwhile, we also noticed that the most closed protein CLP1 from *N. crassa* [10], is reannotated as a cellobionic acid transporter CBT1 [24]. However, in our cellobionic acid uptake test, the yeasts either expressing TRE77517 or CRT1 could not transport cellobionic acid (Fig. 2e).

### Subcellular distribution of TRE77517 in *T. reesei* and its effect on cellulase induction

**Fig. 3:**
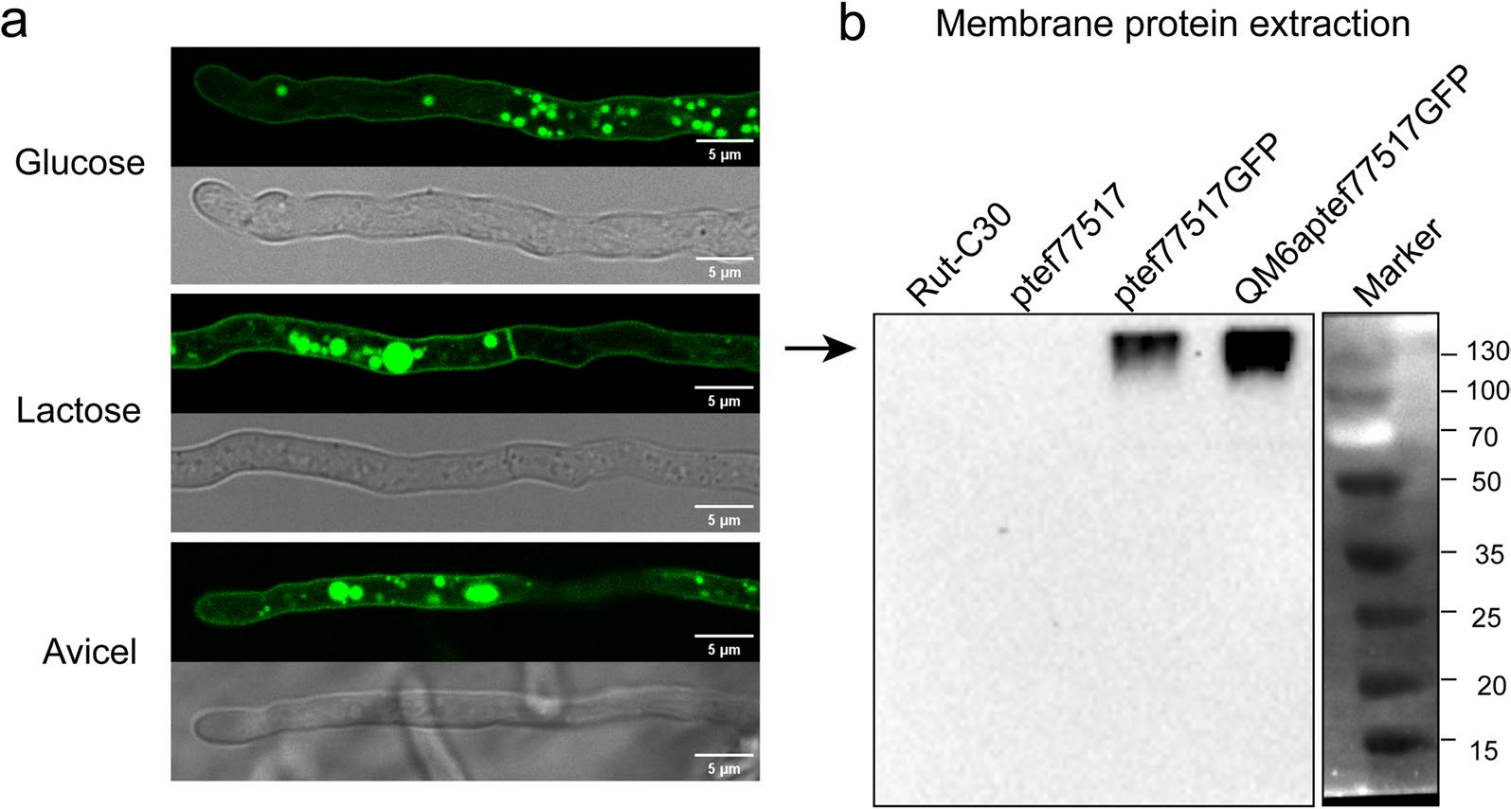
Subcellular distribution of TRE77517GFP in *T. reesei*. **a** C-terminal GFP fused TRE77517 was expressed under the control of strong constitutive promoter Ptef in Rut-C30, resulting in strain ptef77517GFP. ptef77517GFP was cultured in MM medium plus 2 g/L tryptone for 18 h when glucose and lactose was used as carbon source, and for 24 h in Avicel medium. Images were taken under a 63 × oil objective. Scale bar represents 5 μm. **b** Membrane protein extraction from strain Rut-C30, ptef77517, ptef77517GFP and QM6aptef77517GFP were applied for Western blot using an anti-GFP antibody with 1:2000 dilution. Strains were cultured for 18 h in glucose MM medium before membrane protein extraction. Membrane protein extraction was applied as described in methods section. The blot signal of TRE77517GFP was indicated by the arrow. The calculated molecular weight for TRE77517GFP is 522 amino acid + GFP, while the blot signal is higher than expected which might be attributed to post-translational modification

To further study the function of TRE77517 in *T. reesei*, a *tre77517* disrupted strain Δ77517 was generated from Rut-C30. Besides, the constitutive promoter Ptef was used to control the expression of *tre77517* in Rut-C30, resulting in two transformants, ptef77517a and ptef77517b. To study its subcellular location in *T. reesei*, TRE77517GFP which is TRE77517 fused with GFP was expressed in situ; meanwhile, another copy of TRE777517GFP was expressed using its native promoter in *T. reesei*. However, no obvious fluorescence was detected in either transformant even in Avicel medium, which might be attributed to their low-level expression. Then, we successfully found a membrane location of TRE77517GFP using the strong constitutive promoter Ptef on glucose MM medium, which is consistent with its transmembrane region prediction as a membrane protein (Fig. 3a). Unexpectedly, a large part of TRE77517GFP accumulated intracellularly in the vacuoles and endosome compartments. We found that the dual-distribution is still observed when lactose or Avicel was used as carbon source, indicating that this phenomenon is carbon source independent (Fig. 3a). The strains overexpressing TRE77517GFP could improve cellulase induction as that in ptef77517b (Additional file 1: Figure S4), indicated that GFP fusion did not affect the function of TRE77517. The intracellular accumulation of TRE77517GFP in *T. reesei* might be attributed to its high-level expression, which is prone to recycle from membrane and degradation [16]. We also extracted the membrane protein from the strain ptef77517GFP and detected the band of TRE77517GFP in the extraction (Fig. 3b). Although the band of TRE77517GFP is beyond 130 kDa and is largely exceed its expected molecular weight (522 amino acid + GFP), this might attributed to some post-translational modification.

**Fig. 4:**
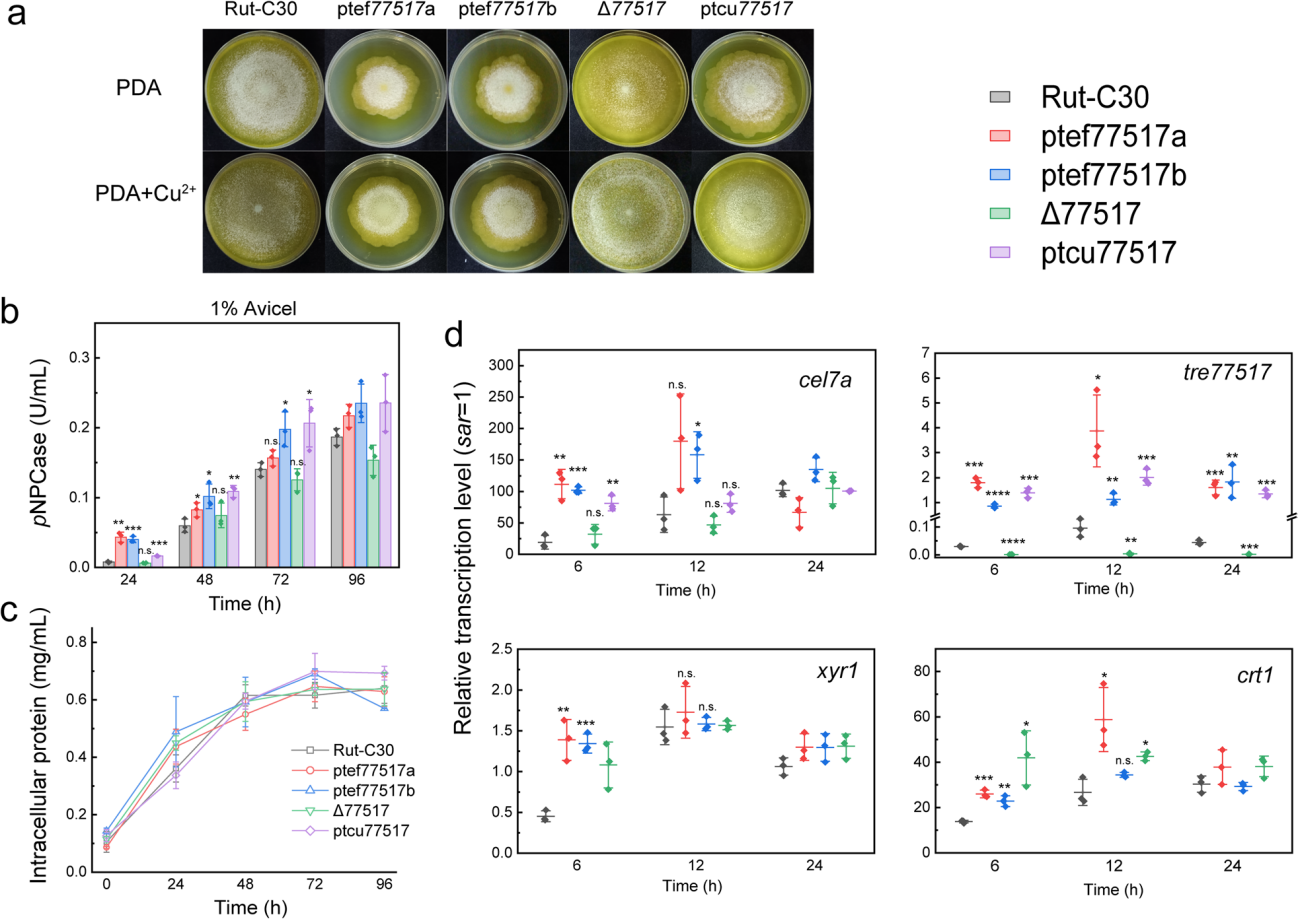
Sugar transporter-like protein TRE77517 regulates cellulase induction. **a** Plate assay of the effect of TRE77517 dysfunction on polarized growth on PDA plates. Approximately 10^4^ conidia of Rut-C30, ptef77517a, ptef77517b, Δ77517 and ptcu77517 were inoculated on the PDA plate and cultured at 30 °C for 5 days before images were taken. 20 μM CuSO_4_ was added as needed. **b** Cellulase production on MM medium with 1% Avicel as the carbon source. Strains were precultured on glucose MM medium plus 2 g/L tryptone for mycelia accumulation, then equal amounts of mycelia were transferred to a fresh MM medium with 10 g/L Avicel as sole carbon source. The *p*NPCase activity was analyzed during fermentation. **c** Mycelia in Avicel medium are represented as intracellular protein content. **d** Mycelia were transferred to Avicel medium, and samples were taken at 6 h, 12 h and 24 h. The transcription level of the major cellulase gene *cel7a*, transcription factor *xyr1*, cellulase response transporter *crt1* and *tre77517* was measured and normalized to the transcription of *sar1*. Values are presented as the mean with the standard deviation from three biological replicates. Statistical significance was calculated through Student’s *t* test. The *p* ≥ 0.05 means no significant. *p* < 0.05 was considered as statistical significance and was indicated as *. *p* < 0.01 was indicated as **. *p* < 0.001 was indicated as ***. *p* < 0.0001 was indicated as ****

The biological function of TRE77517 on fungal growth and cellulase induction was further investigated. The hyphal spread on PDA plate was significantly slower in the *tre77517*-overexpression strains ptef77517a and ptef77517b, while the disruption of *tre77517* did not result in growth defects during the plate assay. A similar phenotype was observed in the strain ptcu77517, in which compact mycelium was observed only in PDA, but it turned to the wild-type-like phenotype when copper was added (Fig. 4a). These results indicated that slower growth in the PDA plate was generated through constitutive expression of *tre77517*.

When cultured on Avicel MM medium, the strains ptef77517a, ptef77517b and ptcu77517 all showed significant improvement in cellulase production in the early stage after the mycelia were transferred to Avicel medium (Fig. 4b, c). However, we found that the disruption of *tre77517* does not affect its cellulase induction (Fig. 4b). The analysis of transcription levels shows a similar pattern. The transcription of *cel7a* was significantly improved in three *tre77517*-overexpression transformants before 12 h, while it returned to the wild-type level at 24 h despite the high-level expression of *tre77517* (Fig. 4d), which might be the reason for the comparative cellulase production from 72 h. Consistent with the cellulase activity in Δ77517, the transcription of *cel7a* in Δ77517 was similar to the level in the parent strain, which further confirmed that the deletion of *tre77517* does not affect cellulase induction on Avicel. Moreover, *tre77517* is lied adjacent to a cellulase activator gene *ace3* (*tre77513*) and an intracellular glucosidase gene *cel1b* (*tre22197*). However, we found that the overexpression of TRE77517 did not improves the expression of these two adjacent genes, while its deletion lead to a slight upregulation under Avicel induction (Additional file 1: Figure S5).

In addition, we also noticed that the transcription of transporter *crt1* and transcription factor *xyr1* in ptef77517a and ptef77517b transformants was improved when the mycelia were exposed to Avicel. However, the transcription of *xyr1* remained at the wild-type level from 12 h, and a similar phenomenon was observed for *crt1* from 24 h (Fig. 4d), consistent with the transcription of *cel7a*, in which the upregulation of *crt1* and *xyr1* in the early induction stage boosted the transcription of the cellulase gene *cel7a*. However, the upregulation of these genes was only observed at the initial stage when mycelia were exposed to Avicel. Surprisingly, we found that the transcription of *crt1* was still upregulated at an early stage in Δ77517 when the mycelia were transferred to Avicel MM medium (Fig. 4d). These data collectively reveal that TRE77517 only affects the gene expression in the early stage of cellulase induction. Then, we speculated that TRE77517 might function in initiating cellulase induction and designated it cellulase response transporter-like protein 2 (CRT2).

### Dysfunction of CRT2 (TRE77517) improves cellulase production without affecting sugar utilization

**Fig. 5:**
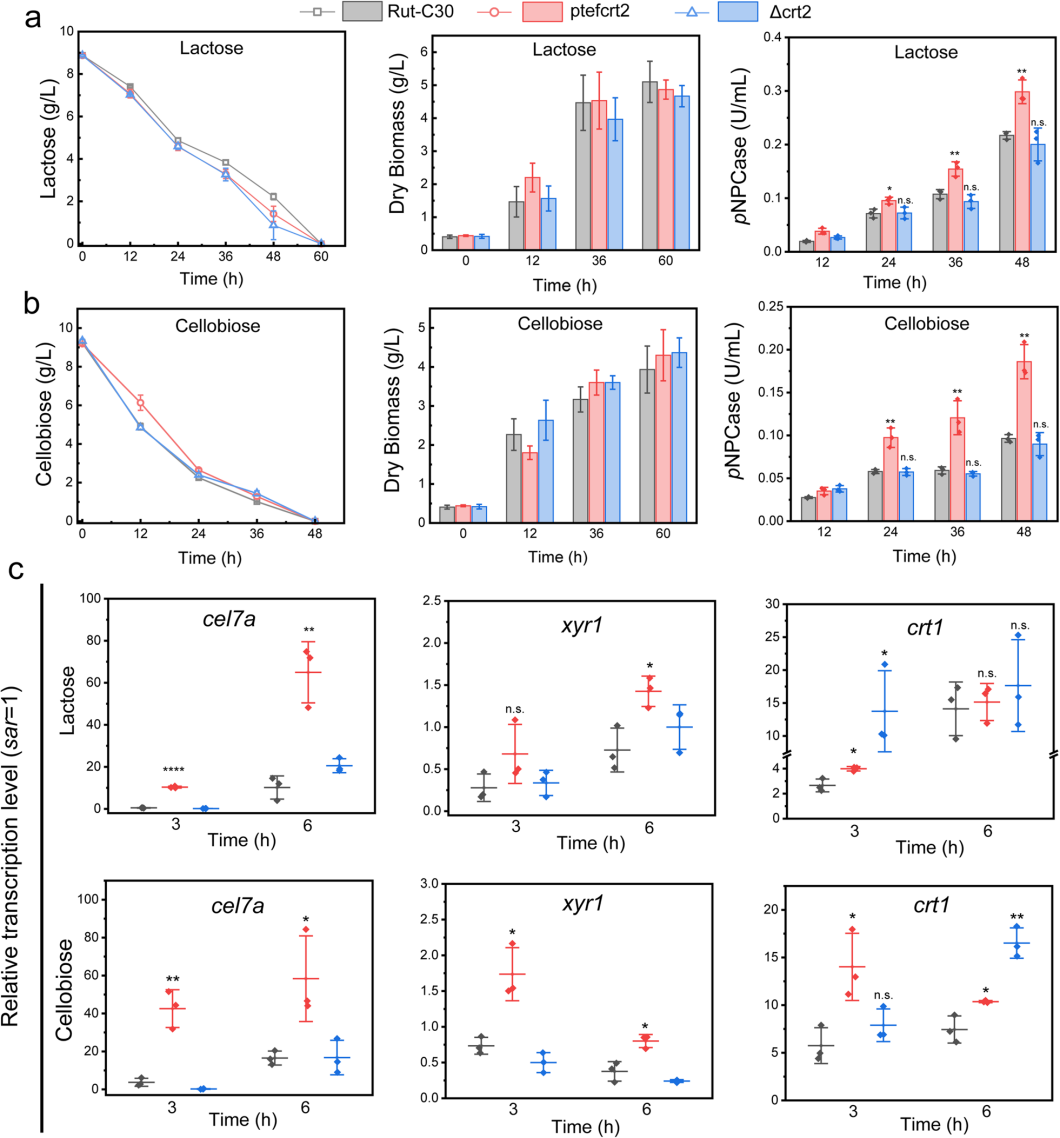
Dysfunction of CRT2 (TRE77517) did not affect cellobiose/lactose consumption. Parent strain Rut-C30, ptefcrt2 and Δcrt2 were precultured on glucose MM medium plus 2 g/L tryptone for 48 h. Equal amounts of pre-grown mycelia were transferred to MM medium with 10 g/L lactose (**a**) or cellobiose (**b**) as sole carbon sources. Samples were taken at indicated time, the residual sugar, dry biomass and cellulase activity were measured. **c** Transcription analysis of cellulase gene *cel7a*, cellobiose/lactose transporter *crt1* and transcription factor *xyr1* in lactose and cellobiose medium. Samples were taken at 3 h and 6 h after mycelia were transferred to the MM medium with lactose or cellobiose as the sole carbon source. All the values represent the mean ± S.D. of three individual experiments. n.s. not significant (p > 0.05), *p < 0.05, **p < 0.01, Student’s *t* test

Although CRT2 did not exhibit the ability to transport sugar in *S. cerevisiae*, we found that either overexpression or deletion of *crt2* (ptefcrt2, Δcrt2) highly activated the expression of *crt1* at the early stage when the mycelia were exposed to Avicel. Meanwhile, a previous study indicated that lactose uptake was impaired in a *crt2* knockout strain [13]; thus, we examined sugar utilization in these strains to understand the role of CRT2 in *T. reesei*. We then used ptef77517b to represent the *crt2* constitutively expressed strain (ptefcrt2) and conducted the following experiments. Equal amounts of pregrown mycelia of ptefcrt2, Δcrt2 and the parent strain were transferred to MM medium with lactose or cellobiose as the sole carbon source; however, these strains did not show significant differences in the utilization of cellobiose or lactose, as well as their biomass accumulation (Fig. 5a, b). Notably, a significant improvement in cellulase production was observed in the strain ptefcrt2, either in the cellobiose or lactose medium (Fig. 5a, b). Deletion of *crt2* does not affect cellulase production on cellobiose and lactose, which was inconsistent with a previous study showing that deletion of *crt2* impaired lactose uptake and cellulase production [13].

To further explore the cellulase response to lactose and cellobiose in ptefcrt2 and Δcrt2, the transcription levels of the cellulase and lactose transporter CRT1 were analyzed in these strains. The transcription of *cel7a* was highly induced in strain ptefcrt2 both in the cellobiose and lactose medium (Fig. 5c), and the transcription of the major regulator *xyr1* and transporter *crt1* were also upregulated when mycelia were exposed to cellobiose and lactose, similar to the phenotype in Avicel medium (Fig. 4d). Following the comparable cellulase production in Δcrt2, the transcription of *cel7a* in Δcrt2 was similar to that in the parent (Fig. 5c). These results indicated that the deletion of *crt2* did not affect cellulase production either in cellobiose or lactose medium. Moreover, we also found that the transcription of the major cellobiose transporter *crt1* was significantly upregulated at the early stage when the mycelia of Δcrt2 were transferred to a lactose or cellobiose medium (Fig. 5c), it turns to the wild-type level after 6 h (Fig. 5c). This might be a feedback activation by deletion of *crt2*, the upregulation of *crt1* at such a short time might not be sufficient to increase the cellobiose uptake. Besides, deletion of the transceptor *clp1* in *N. crassa* Δ3βG background did not affect cellobiose consumption, but activated the expression of *cdt-1* and *cdt-2* [10]. Besides, the upregulated *hgt1/2* and *xyt-1* could compensate for the functional loss of GLT-1 in *N. crassa* [7]. These results suggested that sugar transporters are coordinately regulated.

### CRT2 functions in a CRT1-like manner in cellulase induction by activating CRT1 and XYR1

**Fig. 6:**
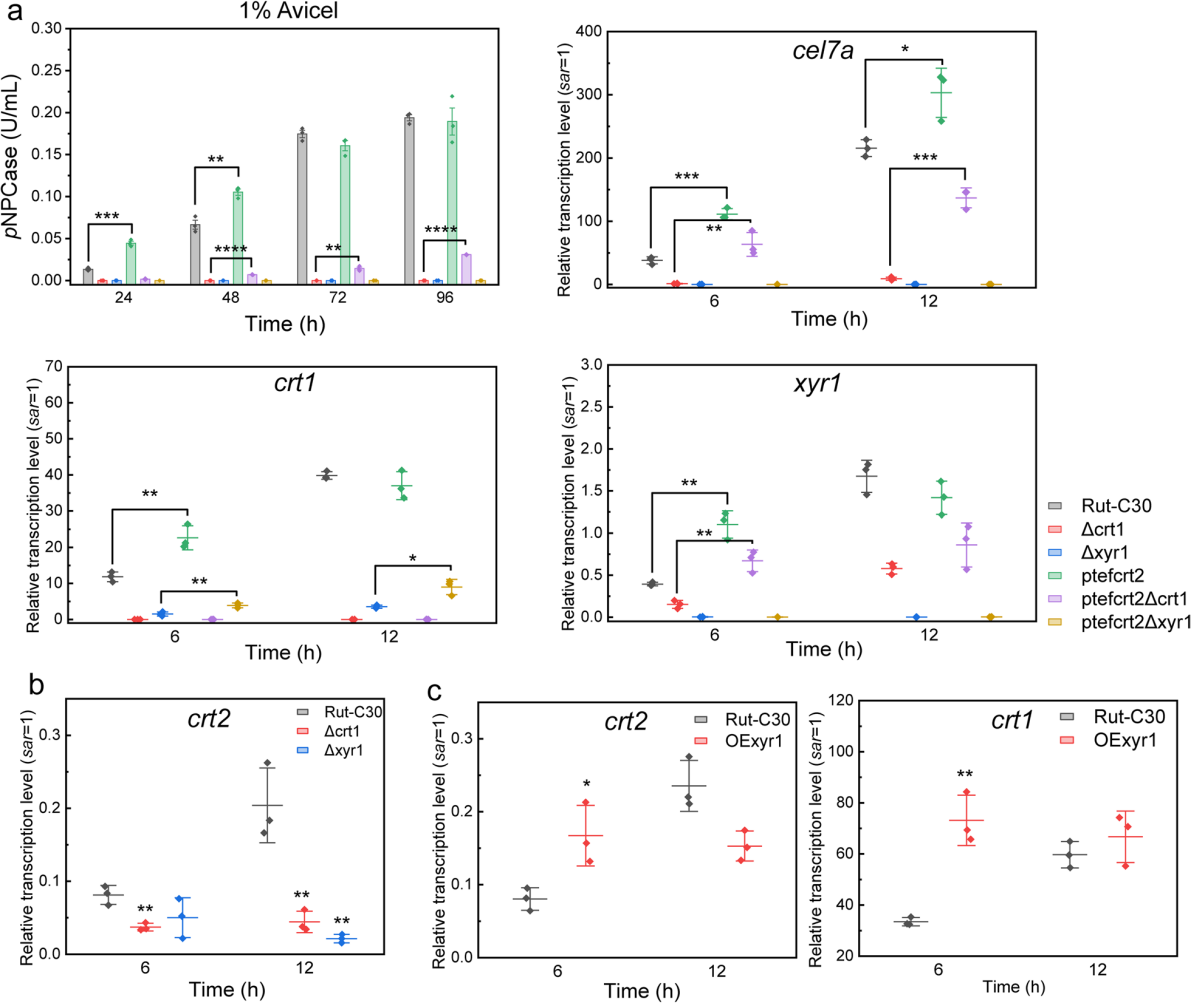
CRT2 functions in a CRT1-like manner in cellulase induction by activating CRT1 and XYR1. **a** Analysis of *p*NPCase activity in Rut-C30, Δcrt1, Δxyr1, ptefcrt2, ptefcrt2Δcrt1 and ptefcrt2Δxyr1 cultured with 1% Avicel for indicated time. Mycelia were collected for transcription analysis after being transferred to Avicel MM medium for 6 h, 12 h. The transcription of *cel7a*, *crt1* and *xyr1* were normalized to the transcription of endogenous *sar1*. **b** Effect of deletion of *crt1* or *xyr1* on *crt2* expression on Avicel medium. **c** Transcription of *crt2* and *crt1* when the major activator XYR1 is overexpressed on Avicel medium. Samples were taken at indicated time and the transcription of *sar1* was set as 1. All the values represent the mean ± S.D. of three individual experiments. *p < 0.05, **p < 0.01, ***p < 0.001, ****p < 0.0001, Student’s *t* test

As previously indicated, the overexpression of *crt2* could activate the expression of the transporter *crt1*, as well as the transcription factor *xyr1*, thus resulting in the upregulation of cellulase. To further characterize the relationship between CRT2, CRT1 and XYR1, we generated *crt1*- and *xyr1*-disrupted strains in the *crt2*-overexpression background. As anticipated, cellulase production was impaired in Δ*crt1* and Δ*xyr1* cultured on Avicel medium (Fig. 6a), consistent with previous studies [14, 25]. Importantly, we found that the constitutive expression of *crt2* in Δcrt1 background (ptefcrt2Δcrt1) partially rescues the cellulase induction on Avicel. The transcriptional analysis confirmed that the expression of *cel7a* is activated in ptefcrt2Δcrt1 (Fig. 6a), although it was slightly weaker than that in Rut-C30. In addition, the transcription of *xyr1* was upregulated in ptefcrt2Δcrt1 compared to the transcription of *xyr1* in Δcrt1, suggesting that the overexpression of Crt2 could activate the expression of *xyr1* even when the cellulase response transporter CRT1 is disrupted (Fig. 6a). Similarly, we found that CRT2-overexpression upregulates the transcription of *crt1* in a XYR1-disrupted background (ptefcrt2Δxyr1), but failed to rescue the *cel7a* expression (Fig. 6a).

Moreover, we found that the expression of *crt2* was downregulated in *crt1* and *xyr1* disruption strains on Avicel medium (Fig. 6b). Besides, the expression of *crt2* on Avicel shows a similar trend to the expression of *crt1*, and *xyr1*-overexpression could activate the expression of *crt2* and *crt1* in the early stage of cellulase induction (Fig. 6c). These data collectively indicated that the function of CRT2 in cellulase induction is similar to that of CRT1, which induce the cellulase expression through activating XYR1, as well as CRT1. However, the induction mediated by CRT2 is less pronounced than CRT1, we speculated that CRT2 only has an auxiliary role by facilitating CRT1 in cellulase induction.

### Activated cellulase production through constitutive expression of *crt2* is still repressed by carbon catabolite repression

**Fig. 7:**
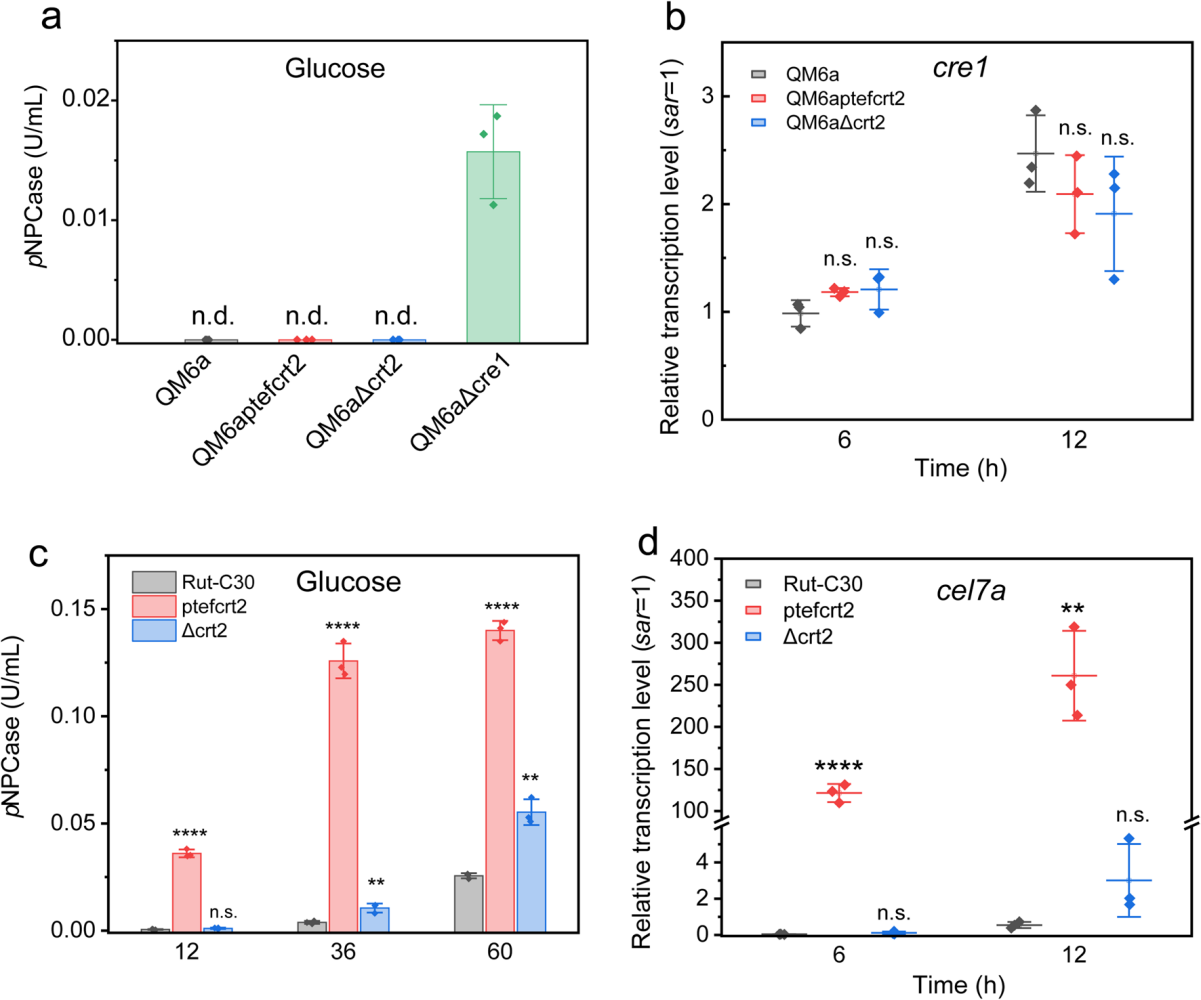
Activated cellulase induction through overexpressed *crt2* was still repressed by CCR. **a** Effect of dysfunction of CRT2 for the cellulase production in QM6a background on glucose medium. Equal amount of precultured QM6a, QM6aptefcrt2, QM6aΔcrt2 and QM6aΔcre1 were transferred to fresh MM medium with 10 g/L glucose as carbon source. *p*NPCase activity was measured after mycelia were transferred for 24 h. n.d. not detected. **b** Transcription of CCR regulator *cre1* on Avicel medium. Precultured mycelia were transferred to Avicel MM medium, samples at 6 h, and 12 h were taken and analyzed for transcription level. **c** Cellulase production by constitutive expression of *crt2* on glucose MM medium in CCR-negative strain Rut-C30. Samples were taken at indicated time and analyzed for *p*NPCase activity (**c**) and transcription level (**d**). All the data were presented as the mean value from three biological replicates. *p* ≥ 0.05 means no significant. *p* < 0.05 was considered as statistical significance and was indicated as *. *p* < 0.01 was indicated as **. *p* < 0.001 was indicated as ***. *p* < 0.0001 was indicated as ****

A previous study indicated that overexpression of the cellobiose transporter CRT1 could slightly relieve the carbon catabolite repression of cellulase production [16]. In our study, the dysfunction of CRT2 could activate the transcription of *crt1*. To study whether the constitutive expression of *crt2* could relieve CCR, we used QM6a as a background, whose cellulase production was fully repressed through CRE1-mediated CCR. When transformants were cultured in glucose MM medium, we found that cellulase production was repressed in QM6aptefcrt2 and QM6aΔcrt2, while the strain QM6aΔcre1 could relieve the repression when glucose was present (Fig. 7a). Likewise, membrane-bound CRT2GFP was observed through microscopy analysis in the QM6a background (Additional file 1: Figure S6), and the constitutive expression of *crt2* could activate the expression of *cel7a*, *crt1* and *xyr1* on Avicel (Additional file 1: Figure S7), indicating that CRT2 works in a similar manner in QM6a. In addition, we found that the expression of the CCR regulator *cre1* was not affected in *crt2* misexpressed strains cultured on Avicel (Fig. 7b), indicating that dysfunction of CRT2 can not affect carbon catabolite repression. Notably, in the Rut-C30 background, which was released from CCR due to the truncated CRE1, the constitutive expression of *crt2* efficiently promoted cellulase production on glucose MM medium (Fig. 7c, d). These results collectively suggested that the activated cellulase production through dysfunction of CRT2 was still repressed by CCR.

## Discussion

Cellulase induction is highly activated by cellulosic substrate in filamentous fungus *T. reesei*. Successive efforts have been made to clarify the mechanism of cellulase induction, including the discovery of the key transcription activators XYR1 [25] and ACE3 [26, 27]. Moreover, some repressors, including CRE1 [28], ACE1 [29] and CTF1 [30], are also involved in the efficient regulation of cellulase production. These transcription factors collectively regulate cellulase expression at the transcription level. Meanwhile, sugar transporters are indispensable for substrate exchange between the cytoplasm and environment and can regulate cellulase induction, probably by transporting inducers or working as signal transceptors [14]. Although 55 sugar transporters have been annotated in the *T. reesei* genome [31], only a few of them have been characterized [16, 19, 32–34]. Among these sugar transporters, the cellobiose/lactose transporter CRT1 is critical for cellulase induction in *T. reesei*, and the disruption of CRT1 impaired cellulase induction either on cellulose or soluble lactose [14–16]. Meanwhile, several major facilitator superfamily permease encoding genes (including CRT1) are highly upregulated in the presence of cellulose-based substrates [18], suggesting that these sugar transporters are collaboratively involved in the perception of cellulose and cellulase synthesis. However, which sugar transporters work in parallel and how these transporters regulate cellulase induction need further exploration. In this study, we characterized a novel cellulose response transporter-like protein CRT2, which is not capable for sugar transport, but shares slight similarity to CRT1 which transduce inducing signal through activating the major cellobiose transporter CRT1 and the transcription factor XYR1.

Previous study conducted by Porciuncula et al. indicated a delayed lactose uptake when *crt2* was disrupted [13], but our study proved that CRT2 cannot transport lactose and cellobiose (Fig. 2d, Additional file 1: Figure S3). Either overexpression or deletion of CRT2 did not affect the cellobiose and lactose utilization in *T. reesei* (Fig. 5a, b). The possible reasons for such conflicting results might be the differences in the inoculation method (spores by Porciuncula et al. and pre-grown mycelia in this study) and the strain background.

CRT2GFP locates in the membrane of *T. reesei*, which is consistent with its transmembrane region prediction, but we still found some parts of CRT2GFP accumulate in the intracellular compartments, which mainly in vacuoles and endolytic vesicles (Fig. 3a), and similar results were obtained in the wild type *T. reesei* QM6a (Additional file 1: Figure S6). In addition, the GFP fusion to CRT2 did not affect the function of CRT2 (Additional file 1: Figure S4). Several sugar transporters have been found to be internalized to transduce cellular signal intracellularly, such as the N-acetylglucosamine transporter NGT1 in *Candida albicans*. NGT1 undergoes continuous internalization when exposed to N-acetylglucosamine (GlcNAc) and trafficks from membrane towards vacuoles [35]. Besides, the cellobiose transporter CRT1 in *T. reesei* is localized at the cell membranes as well as at the periphery of the nucleus [16]. In our study, the membrane CRT2 also locates in vacuoles probably due to overexpression of *crt2* in the strain ptefcrt2GFP. The expression of *crt2* using the strong promoter Ptef is nearly 30-fold higher than its expression in the parent strain (Fig. 4d). The vacuolar distribution of CRT2GFP might be attributed to its high-level expression that could recycle from membrane to avoid the permanent membrane occupancy as indicated previously [16]. Overall, our data confirmed that CRT2 acts as a membrane protein in *T. reesei*, but whether the intracellular accumulation of CRT2GFP is functional relevance needs further investigation.

**Fig. 8:**
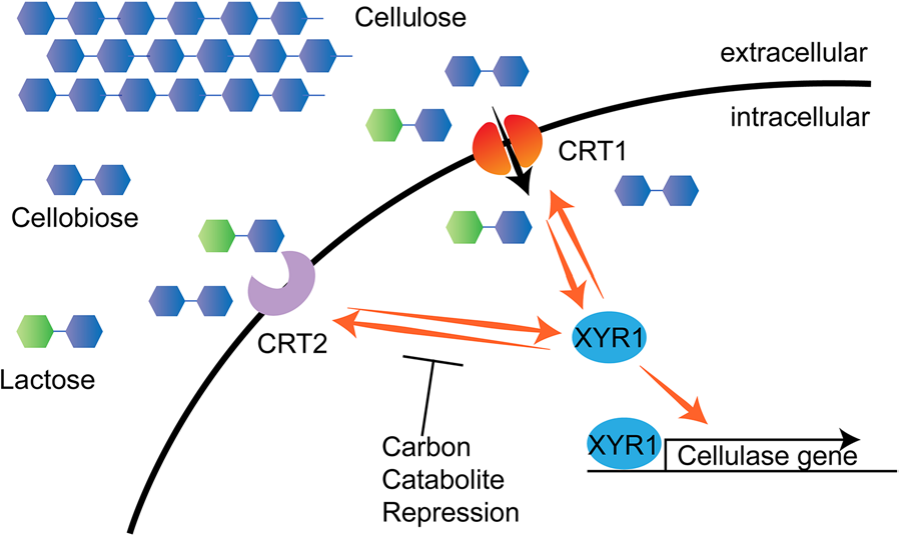
Diagram of the cellulase induction by CRT2, CRT1 and XYR1 in *T. reesei*. When cellulose, cellobiose or lactose are used as carbon sources, the expression of *crt2* and *crt1* is activated. The cellulase induction signal is transduced by CRT1 and CRT2, and activate the expression of major transcription activator XYR1, which finally induce the cellulase production. Whereas the expression of *crt2* is also under the tight regulation by XYR1 in the cellulase induction. However, the activated cellulase production by constitutive expression of *crt2* cannot antagonize glucose-mediated CCR

The cellobiose transporter CRT1 is indispensable for cellulase induction in *T. reesei* [14]. In our study, we found that CRT2 functions in a similar manner as CRT1 in cellulase induction. The speculative model of the role of CRT2 in the regulation of cellulase induction is as follows (Fig. 8): (i) both CRT1 and CRT2 are induced by Avicel, lactose and cellobiose (Additional file 1: Figure S8) [13, 17]. (ii) CRT1 acts as a signal transductor and activates the major transcription factor XYR1, which further activates the cellulase expression. CRT2 could also activate XYR1 during cellulase induction. (iii) Both CRT1 and CRT2 are under the positive regulation of XYR1 in the early stage of cellulase induction (Figs. 6c, 8). Meanwhile, it is notable that *crt2* is expressed in a 300-fold lower level compared to that of *crt1* (Fig. 6c), and the cellulase activation by CRT2-overexpressing is moderate compared to CRT1 (Fig. 6a), suggesting that CRT2 may have an auxiliary role in cellulase induction, and is less critical than CRT1. A recent study proved that overexpression of XYR1 fully rescued the defect in cellulase induction in strain Δcrt1 [16]. In this study, the expression of *xyr1* is upregulated in Δcrt1 background when CRT2 is overexpressed, indicating that CRT2 might transduce the inducing signal to activate XYR1. The upregulated *xyr1* in ptefcrt2Δcrt1 compared to Δcrt1 further rescued the cellulase production (Fig. 6a).

In general, fungal sugar transporters take part in substrate exchange and sugar transport, but some are also involved in signal transduction [3, 5, 10, 14, 15]. Some post-translational modifications on sugar transporters such as phosphorylation and ubiquitination of their C-terminal tail are important for their signal transduction [36, 37]. The glucose sensor RGT2 in *S. cerevisiae* has a similar structure to those glucose transporters *HXTs*, but is unable to transport glucose. The phosphorylation on the 220 amino acid C-terminus of RGT2 is critical for the interaction with other regulators and successive activation of glucose transporters [37, 38]. The C-terminus of cellobiose transporter CRT1 is evidenced for its signal transduction, which is separable from the lactose transport capacity [16]. Although no phosphorylation sites and ubiquitination consensus were predicted in the 44-amino acid C-terminal tail of CRT1 [15, 16], truncation of the C-terminus with only five amino acids left fully impaired its cellulase induction. This indicates that other uncharacterized consensus in CRT1 are crucial for its signal transduction. Moreover, expression of a cellobiose transporter CDT-1 from *N. crassa* or LacpB from *A. nidulans* rescue the cellulase induction defect in Δcrt1 background [15], and these sugar transporters also share similar sequences (Fig. 2b), which is the basis of highly similar functions. CRT2 has a similar structure to CRT1 but could not transport cellobiose and lactose. The C-terminus of CRT2 harbors two phosphorylation sites at T471 and T506 (https://scansite4.mit.edu/#home) [39], and a potential ubiquitin site at K514 (http://gpsuber.biocuckoo.cn/) [40]. Thus, we speculate that CRT2 works as a signal transductor for cellulose/cellobiose perception (Fig. 8), similar to the pattern of RGT2 in *S. cerevisiae* [37]. The C-terminus of CRT2 might be engaged in signal transduction through potential phosphorylation and ubiquitination, which is consistent with the oversized band in our western blot assay (Fig. 3b), but the exact mechanism and other regulators involved still need further study.

## Conclusion

In this study, we give a detailed functional analysis of a previously annotated putative lactose permease CRT2 (TRE77517). CRT2 cannot transport lactose and cellobiose, and neither the deletion or overexpression of CRT2 affects the cellobiose and lactose uptake in *T. reesei*. CRT2 is proven to be a membrane protein and is involved in cellulase induction by activating the major transcription factor XYR1 and cellobiose transporter CRT1. CRT2 overexpression improves cellulase induction either on Avicel, cellobiose or lactose medium, and could partially rescue the cellulase induction defect in *crt1*-deleted strain. Thus, we speculated that CRT2 acts as a sugar transporter-like signal transductor to facilitate CRT1 in cellulase induction. This study gives novel insights of sugar transporters in cellulase induction, expanding the understanding of the mechanism in cellulase induction.

## Methods

### Strains and culture conditions

*Escherichia coli* JM109 was cultivated in 37 °C for plasmid construction. *Agrobacterium tumefaciens* AGL1 was used for fungal transformation. *T. reesei* Rut-C30 and QM6a was used as parent strains and maintained on PDA plate at 30 °C. For liquid cultivation, 1 × 10^7^ conidia were inoculated into 50 mL MM liquid medium (15 g/L KH_2_PO_4_, 5 g/L (NH_4_)_2_SO_4_, 0.6 g/L CaCl_2_, 0.6 g/L MgSO_4_, 0.005 g/L FeSO_4_*7H_2_O, 0.0012 g/L MnSO_4_*H_2_O, 0.0014 g/L ZnSO_4_*7H_2_O, 0.002 g/L CoCl_2_*6H_2_O, pH 5.2) containing 10 g/L glucose and 2 g/L tryptone for better mycelia accumulation. After 48 h cultivation at 28 °C 200 rpm, equal amounts of mycelia were transferred to a new MM liquid medium with 1% indicated carbon sources. Samples were taken at indicated time, the mycelia were collected for RT-qPCR or biomass determination, and the supernatant was used for biochemistry analysis. For initial test the effect of sugar transporter for cellulase production in Fig. 1, 0.5 × 10^7^ conidia was inoculated into 25 mL SDB broth (40 g/L glucose, 10 g/L yeast extract and 10 g/L tryptone) for 40 h, and equal amounts of mycelia was transferred to 50 mL Avicel inducing medium (20 g/L Avicel, 4 g/L KH_2_PO_4_, 2.8 g/L (NH_4_)_2_SO_4_, 3 g/L tryptone, 0.6 g/L Urea, 0.1%(v/v) Tween 80, 0.6 g/L CaCl_2_, 0.6 g/L MgSO_4_, 0.005 g/L FeSO_4_*7H_2_O, 0.0012 g/L MnSO_4_*H_2_O, 0.0014 g/L ZnSO_4_*7H_2_O, 0.002 g/L CoCl_2_*6H_2_O, 5 g/L CaCO_3_, pH 5.5) with 28 °C 200 rpm for indicated time. *Saccharomyces cerevisiae* hexose-transport null mutant EBY.VW4000 was used as host for analyzing the sugar transport of Tre77517. Yeast strains were maintained at 30 °C in SC medium (6.7 g/L yeast nitrogen base, 1.85 g/L drop out mixture without histidine and uracil) with 20 g/L desired carbon sources.

### Plasmid construction and strains transformation

To construct the promoter replacing plasmid, approximate -2000 bp ~ -800 bp of ATG was used as upstream flank, about 1200 bp from ATG was set as downstream flank. Recombinant flanks were amplified from genomic DNA of *T. reesei.* The *hyg* or *ura3* selection marker was amplified from previous constructed plasmid pCAMBIA1301G [41]. Ptcu promoter is cloned from genomic DNA from Rut-C30. For each promoter replacing plasmid, the up/down-stream fragments, Ptcu promoter and selection marker were assembled into the basic backbone of pCAMBIA1301G, which contains necessary element for *Agrobacterium tumefaciens* mediated transformation using the MultiF SeamLess Assembly Mix (Abclonal, Wuhan, China). Briefly, the molecular ratio for upstream flank: downstream flank: Ptcu promoter: selection marker: backbone is 3:3:3:3:1, all the fragments were added to a final volume of 5 μL, thereafter the 5 μL Assembly Mix was added and incubated at 50 °C for 30 min. The resultant products were used for *E. coli* transformation.

The *crt2* (*tre77517*) overexpression cassette was constructed using the 800 bp Ptef promoter, which was amplified from genomic DNA of *T. reesei*. The coding region of *crt2* was amplified from the cDNA of Rut-C30 exposed to Avicel for 12 h. The amplified fragments were assembled with the Tcbh1 terminator, resulting in *Ptef-crt2-Tcbh1* cassette, and fused with the URA3 selection marker to the backbone of pCAMBIA1301G using the MultiF SeamLess Assembly Mix. To knockout *crt2*, approximate 1200 bp upstream and downstream flank of *crt2* was assembled with URA3 selection marker and backbone. The knockout cassette for *xyr1*, *crt1* and *cre1* were constructed in a similar manner with *hyg* selection marker. For analysis of the subcellular localization of CRT2 in *T. reesei*, the strong constitutive promoter Ptef was used to drive the expression of *crt2*. *gfp* fragment was fused followed by a ‘GGGS’ linker. These fragments were assembled with *hyg* selection marker and backbone from pCAMBIA1301G to form ptefcrt2GFP plasmid. These plasmids were transformed to Rut-C30 or QM6a as desired purposes using the *Agrobacterium tumefaciens* mediated transformation. The correct transformants were verified by anchor PCR.

To analyze the sugar transport in *S. cerevisiae*, the coding sequence of *tre77517* was synthesized according to the codon usage of *S. cerevisiae*, and ligated to the *EcoR* I and *BamH* I site of pYX212, a commercial available plasmid contains URA3 selection marker, yeast 2μ plasmid origin of replication. The expression of Tre77517 in *S. cerevisiae* was controlled by its constitutive promoter Ptpi. GFP-tagged Tre77517 was also constructed to visualize the subcellular location of Tre77517 in *S. cerevisiae*. Moreover, the coding sequence of Crt1 was amplified from the cDNA of *T. reesei* using the annotation in Rut-C30. It was also inserted in the *EcoR* I and *BamH* I site of pYX212. All these plasmids were transformed into EBY/*gh1-1* which harbored an intracellular β-glucosidase gene *gh1-1* (NCU00130) from *N. crassa*. All the strains, plasmids and primers used in this study were listed in Additional file 1: Tables S1 and S2.

### Enzyme analysis and growth assay

The Filter paper activity (FPase) was measured using DNS method described previously [41], one unit of enzyme activity was defined as the amount of enzyme that release 1 μmol reducing sugar per minute. The cellobiohydrolase activity was determined with 4 mM *p*NPC (*p*-nitrophenyl-β-D-cellobioside) as substrate in a 50 mM NaAc Buffer, pH 5.0. One unit of enzyme activity was defined as the amount of enzyme that releases 1 μmol *p*NP per minute.

The Intracellular protein concentration was measured to represent the biomass in Avicel medium according to our previous study [20]. To measure the biomass accumulation in MM medium with cellobiose and lactose as carbon sources, 1 mL sample were taken and the mycelia were washed twice and dried at 80 °C for 48 h.

For yeast growth analysis, yeast transformants were pre-cultured in SC liquid medium with maltose as carbon source for 16 h. Cells were harvested through centrifugation, and diluted to OD_600_ = 0.5. tenfold serial dilution was applied for each transformant. 5 μL suspension was dropped into SC plate with different carbon sources and cultured at 30 °C for indicated time.

### Phylogenetic analysis

The amino acid sequences of characterized sugar transporters were obtained according to the references. The alignments were conducted by ClustalW, the phylogenetic tree was generated via MEGA-X software (Version 10.2.5) using the neighbor-joining method with 1000 bootstrap replications.

### Sugar analysis

For analyzing the residue lactose and cellobiose in the supernatant, samples were collected and boiled, and then filtered after centrifugation. The sugar concentration was analyzed through HPLC with Bio-Rad Aminex HPX-87H column and Agilent 1260 Infinity II equipped with refractive index detector. 10 μL sample was injected with 5 mM H_2_SO_4_ as mobile phase at a flow rate of 0.5 mL/min.

### Synthesis of cellobionic acid

The synthesis of cellobionic acid is conducted using a mild oxidation method as reported previously [42, 43]. Briefly, 3.8 g cellobiose was dissolved in 20 mL deionized water. The mixture was then poured into 50 mL methanol. 5.7 g iodine was added in 5 mL methanol and then added in the cellobiose–methanol solution, following a constant stir at 40 °C for 15 min. 100 mL of 4% (w/v) KOH–methanol solution was slowly added to the cellobiose–iodine solution with stir in 15 min, forming a sticky yellow precipitate. Another 100 mL of 4% (w/v) KOH–methanol solution was slowly added again and stirred for another 30 min. The precipitate was dissolved with 5 mL water and added to another 100 mL methanol. The white precipitate was collected and washed three times with methanol, residual methanol was removed from products by vacuum drying.

### Sugar uptake assay and HPAEC analysis

Sugar uptake assay is based on the method by Li et al. [24]. Yeasts expressing either empty plasmid pYX212, TRE77517 and CRT1 were cultured in SC medium with maltose as carbon sources lacking uracil and histidine to late log phase. Cells were harvested and washed three times with an assay Buffer (5 mM MES, 100 mM NaCl, pH 6.0) and resuspended to OD_600_ = 30. Different sugars were prepared to a concentration of 200 μM in the assay Buffer. The uptake was initiated by mixed equal volume of yeast strains and sugar, the reaction thereafter was incubated at 30 °C at 200 rpm for 40 min. Samples were centrifuged and filtered before HPAEC analysis.

HPAEC was conducted in ICS5000 + (Thermo Scientific) using a Dionex CarboPac Analytical PA200 (3 × 250 mm) and a Dionex CarboPac Guard PA200 (3 × 50 mm) at 30 °C. 10 μl sample was injected in a 200 mM NaOH mobile phase with 0.4 mL/min. The separation of cellobionic acid is conducted with a sodium acetate gradients from 0 to 500 mM.

### Microscope visualization

*S. cerevisiae* transformants were cultured in SC medium with 20 g/L maltose as carbon source for 16 h, cells were harvested and washed twice, then observed under laser scanning confocal microscope TCS SP8 (Leica, Germany) with 63 × oil immersion objective. *T. reesei* transformants were cultured in MM medium with 10 g/L glucose and 2 g/L tryptone for 18 h, the mycelia were washed before Microscope analysis. All the images were processed through ImageJ 1.53c.

### Membrane protein extraction and Western blot

*T. reesei* strains were cultured in MM medium with 10 g/L glucose and 2 g/L tryptone at 28 °C for 18 h. Mycelia were collected through filtration, the membrane protein extraction was applied using a Membrane Protein Extraction Kit (C500049, Sangon Biotech, Shang Hai, China). The membrane protein is collected in lower organic phase, and followed by precipitation with acetone. The pellet was collected through centrifugation and residual acetone was removed through volatilization on ice. The membrane protein was dissolved in 1 × SDS loading buffer and boiled for 5 min before SDS–PAGE. Western blot was carried out using a 0.45 μm PVDF membrane with standard protocol, the detection of Tre77517GFP was using Anti-GFP mouse monoclonal antibody (D191040, Sangon Biotech, Shang Hai, China) with 1:2000 dilution.

### RNA isolation and RT-qPCR

For RNA isolation, 1 mL sample at indicated time were collected and frozen in liquid nitrogen, stored at −80 °C before use. RNA isolation was conducted using Trizol (Sangon Biotech (Shanghai)) as indicated by user’s guide. HiScript III RT SuperMix for qPCR (+ gDNA wiper) (Vazyme, Nanjing, China) was used for reverse transcription and ChamQ Universal SYBR qPCR Master Mix (Vazyme, Nanjing, China) was applied for RT-qPCR reaction in this study. The transcription level of endogenous *sar1* was set as 1, and the relative transcription level of desired genes was calculated via ΔΔCT method.

### Statistics analysis

All the results were shown as the mean value of three biological replicates (generally three flasks for one strain) except for other indications. The analysis of variance was performed by use of *t* test procedures. A *p* value of *p* < 0.05(*), *p* < 0.01(**), *p* < 0.001(***) or *p* < 0.0001(****) were considered as significant.

## Supplementary Information

**Additional file 1: Figure S1.** Transcription level of sugar transporters during cellulase induction on Avicel inducing medium. **Figure S2.** Analysis of the expression of Ptcu controlled transporters. **Figure S3.** Sugar transport analysis of TRE77517 for other mono/disaccharides. **Figure S4.** Plate assay and cellulase induction by overexpressing TRE77517GFP in *T. reesei*. **Figure S5.** Transcription of the two adjacent gene *ace3* and *cel1b* during cellulase induction. **Figure S6.** Subcellular location of CRT2GFP (TRE77517GFP) in QM6a. **Figure S7.** Dysfunction of CRT2 (TRE77517) affect cellulase induction in the wild type QM6a. **Figure S8.** Expression of *crt2* (*tre77517*) was activated in cellulase-induced carbon sources. **Table S1.** Strains and plasmids used in this study. **Table S2.** Primers used in this study.

## Data Availability

The authors declare that all data supporting the findings of this study are available within the paper and its supplementary information files.

## References

[1] Gupta VK, Steindorff AS, de Paula RG (2016). The post-genomic era of *Trichoderma reesei*: What's next?. Trends Biotechnol.

[2] Yan S, Xu Y, Yu X-W (2021). From induction to secretion: a complicated route for cellulase production in *Trichoderma reesei*. Bioresour Bioprocess.

[3] Nogueira KMV, Mendes V, Carraro CB (2020). Sugar transporters from industrial fungi: key to improving second-generation ethanol production. Renew Sust Energ Rev.

[4] Kubicek CP, Mikus M, Schuster A (2009). Metabolic engineering strategies for the improvement of cellulase production by *Hypocrea jecorina*. Biotechnol Biofuels.

[5] Znameroski EA, Li X, Tsai JC (2014). Evidence for transceptor function of cellodextrin transporters in *Neurospora crassa*. J Biol Chem.

[6] Ozcan S, Johnston M (1999). Function and regulation of yeast hexose transporters. Microbiol Mol Biol Rev.

[7] Wang B, Li J, Gao J (2017). Identification and characterization of the glucose dual-affinity transport system in *Neurospora crassa*: pleiotropic roles in nutrient transport, signaling, and carbon catabolite repression. Biotechnol Biofuels.

[8] Li J, Liu Q, Li J (2021). RCO-3 and COL-26 form an external-to-internal module that regulates the dual-affinity glucose transport system in *Neurospora crassa*. Biotechnol Biofuels.

[9] Galazka JM, Tian C, Beeson WT (2010). Cellodextrin transport in yeast for improved biofuel production. Science.

[10] Cai P, Wang B, Ji J (2015). The putative cellodextrin transporter-like protein CLP1 is involved in cellulase induction in *Neurospora crassa*. J Biol Chem.

[11] Chaudhary N, Kumari I, Sandhu P (2016). Proteome scale census of major facilitator superfamily transporters in *Trichoderma reesei* using protein sequence and structure based classification enhanced ranking. Gene.

[12] Ramos AS, Chambergo FS, Bonaccorsi ED (2006). Oxygen-and glucose-dependent expression of Trhxt1, a putative glucose transporter gene of *Trichoderma reesei*. Biochem.

[13] Porciuncula JD, Furukawa T, Shida Y (2013). Identification of major facilitator transporters involved in cellulase production during lactose culture of *Trichoderma reesei* PC-3-7. Biosci Biotech Bioch.

[14] Zhang WX, Kou YB, Xu JT (2013). Two major facilitator superfamily sugar transporters from *Trichoderma reesei* and their roles in induction of cellulase biosynthesis. J Biol Chem.

[15] Havukainen S, Valkonen M, Koivuranta K (2020). Studies on sugar transporter CRT1 reveal new characteristics that are critical for cellulase induction in *Trichoderma reesei*. Biotechnol Biofuels.

[16] Wang Z, Yang R, Lv W (2022). Functional characterization of sugar transporter CRT1 reveals differential roles of its C-Terminal region in sugar transport and cellulase induction in *Trichoderma reesei*. Microbiol Spectr.

[17] Havukainen S, Pujol-Gimenez J, Valkonen M (2021). Electrophysiological characterization of a diverse group of sugar transporters from *Trichoderma reesei*. Sci Rep.

[18] de Paula RG, Antonieto ACC, Ribeiro LFC (2018). New genomic approaches to enhance biomass degradation by the industrial fungus *Trichoderma reesei*. Int J Genomics.

[19] Nogueira KMV, de Paula RG, Antonieto ACC (2018). Characterization of a novel sugar transporter involved in sugarcane bagasse degradation in *Trichoderma reesei*. Biotechnol Biofuels.

[20] Yan S, Xu Y, Tao XM (2023). Alleviating vacuolar transport improves cellulase production in *Trichoderma*
*reesei*. Appl Microbiol Biotechnol.

[21] Lv X, Zheng F, Li C (2015). Characterization of a copper responsive promoter and its mediated overexpression of the xylanase regulator 1 results in an induction-independent production of cellulases in *Trichoderma reesei*. Biotechnol Biofuels.

[22] Dos Reis TF, de Lima PB, Parachin NS (2016). Identification and characterization of putative xylose and cellobiose transporters in *Aspergillus nidulans*. Biotechnol Biofuels.

[23] Fekete E, Orosz A, Kulcsar L (2016). Characterization of a second physiologically relevant lactose permease gene (lacpB) in *Aspergillus nidulans*. Microbiology (Reading).

[24] Li X, Chomvong K, Yu VY (2015). Cellobionic acid utilization: from Neurospora crassa to Saccharomyces cerevisiae. Biotechnol Biofuels.

[25] Stricker AR, Grosstessner-Hain K, Wurleitner E (2006). Xyr1 (xylanase regulator 1) regulates both the hydrolytic enzyme system and D-xylose metabolism in *Hypocrea jecorina*. Eukaryot Cell.

[26] Häkkinen M, Valkonen MJ, Westerholm-Parvinen A (2014). Screening of candidate regulators for cellulase and hemicellulase production in *Trichoderma reesei* and identification of a factor essential for cellulase production. Biotechnol Biofuels.

[27] Zhang J, Chen Y, Wu C (2019). The transcription factor ACE3 controls cellulase activities and lactose metabolism via two additional regulators in the fungus *Trichoderma reesei*. J Biol Chem.

[28] Ilmen M, Thrane C, Penttila M (1996). The glucose repressor gene *cre1* of *Trichoderma*: isolation and expression of a full-length and a truncated mutant form. Mol Gen Genet.

[29] Aro N, Ilmen M, Saloheimo A (2003). ACEI of *Trichoderma reesei* is a repressor of cellulase and xylanase expression. Appl Environ Microbiol.

[30] Meng QS, Zhang F, Liu CG (2020). Identification of a novel repressor encoded by the putative gene *ctf1* for cellulase biosynthesis in *Trichoderma reesei* through artificial zinc finger engineering. Biotechnol Bioeng.

[31] Martinez D, Berka RM, Henrissat B (2008). Genome sequencing and analysis of the biomass-degrading fungus *Trichoderma*
*reesei* (syn. *Hypocrea*
*jecorina*). Nat Biotechnol.

[32] Xu W, Fang Y, Ding M (2022). Elimination of the sugar transporter GAT1 increased xylanase I production in *Trichoderma reesei*. Front Microbiol.

[33] Havukainen S, Pujol-Gimenez J, Valkonen M (2021). Functional characterization of a highly specific L-arabinose transporter from *Trichoderma reesei*. Microb Cell Fact.

[34] Sloothaak J, Tamayo-Ramos JA, Odoni DI (2016). Identification and functional characterization of novel xylose transporters from the cell factories *Aspergillus niger* and *Trichoderma reesei*. Biotechnol Biofuels.

[35] Hanumantha Rao K, Roy K, Paul S (2022). N-acetylglucosamine transporter, Ngt1, undergoes sugar-responsive endosomal trafficking in *Candida albicans*. Mol Microbiol.

[36] Yoshinari A, Hosokawa T, Beier MP (2021). Transport-coupled ubiquitination of the borate transporter BOR1 for its boron-dependent degradation. Plant Cell.

[37] Moriya H, Johnston M (2004). Glucose sensing and signaling in *Saccharomyces cerevisiae* through the Rgt2 glucose sensor and casein kinase I. Proc Natl Acad Sci U S A.

[38] Snowdon C, Johnston M (2016). A novel role for yeast casein kinases in glucose sensing and signaling. Mol Biol Cell.

[39] Obenauer JC, Cantley LC, Yaffe MB (2003). Scansite 2.0: proteome-wide prediction of cell signaling interactions using short sequence motifs. Nucleic Acids Res.

[40] Wang C, Tan X, Tang D (2022). GPS-Uber: a hybrid-learning framework for prediction of general and E3-specific lysine ubiquitination sites. Brief Bioinform.

[41] Yan S, Xu Y, Yu XW (2021). Rational engineering of xylanase hyper-producing system in *Trichoderma reesei* for efficient biomass degradation. Biotechnol Biofuels.

[42] Hildebrand A, Addison JB, Kasuga T (2016). Cellobionic acid inhibition of cellobiohydrolase I and cellobiose dehydrogenase. Biochem Eng J.

[43] Forsberg Z, Vaaje-Kolstad G, Westereng B (2011). Cleavage of cellulose by a CBM33 protein. Protein Sci.

