# Supplementary material for: Role of cellulose response transporter-like protein CRT2 in cellulase induction in Trichoderma reesei
Source: Biotechnol Biofuels Bioprod. 2023 Jul 24;16:118. doi: 10.1186/s13068-023-02371-7 (PMC10364367; doi:10.1186/s13068-023-02371-7)
Supplement: Supplementary file 1 — Additional file 1: Figure S1. Transcription level of sugar transporters during cellulase induction on Avicel inducing medium. Figure S2. Analysis of the expression of Ptcu controlled transporters. Figure S3. Sugar transport analysis of TRE77517 for other mono/disaccharides. Figure S4. Plate assay and cellulase induction by overexpressing TRE77517GFP in T. reesei. Figure S5. Transcription of the two adjacent gene ace3 and cel1b during cellulase induction. Figure S6. Subcellular location of CRT2GFP (TRE77517GFP) in QM6a. Figure S7. Dysfunction of CRT2 (TRE77517) affect cellulase induction in the wild type QM6a. Figure S8. Expression of crt2 (tre77517) was activated in cellulase-induced carbon sources. Table S1. Strains and plasmids used in this study. Table S2. Primers used in this study. [file 13068_2023_2371_MOESM1_ESM.docx]

**Additional file**

**Role of cellulose response transporter-like protein CRT2 in cellulase induction in *Trichoderma reesei***

Su Yan, Yan Xu, Xiao-Wei Yu*

Lab of Brewing Microbiology and Applied Enzymology, School of Biotechnology and Key Laboratory of Industrial Biotechnology of Ministry of Education, Jiangnan University, Wuxi 214122, China

*Address correspondence to Xiao-Wei Yu, [yuxw@jiangnan.edu.cn](mailto:yuxw@jiangnan.edu.cn)


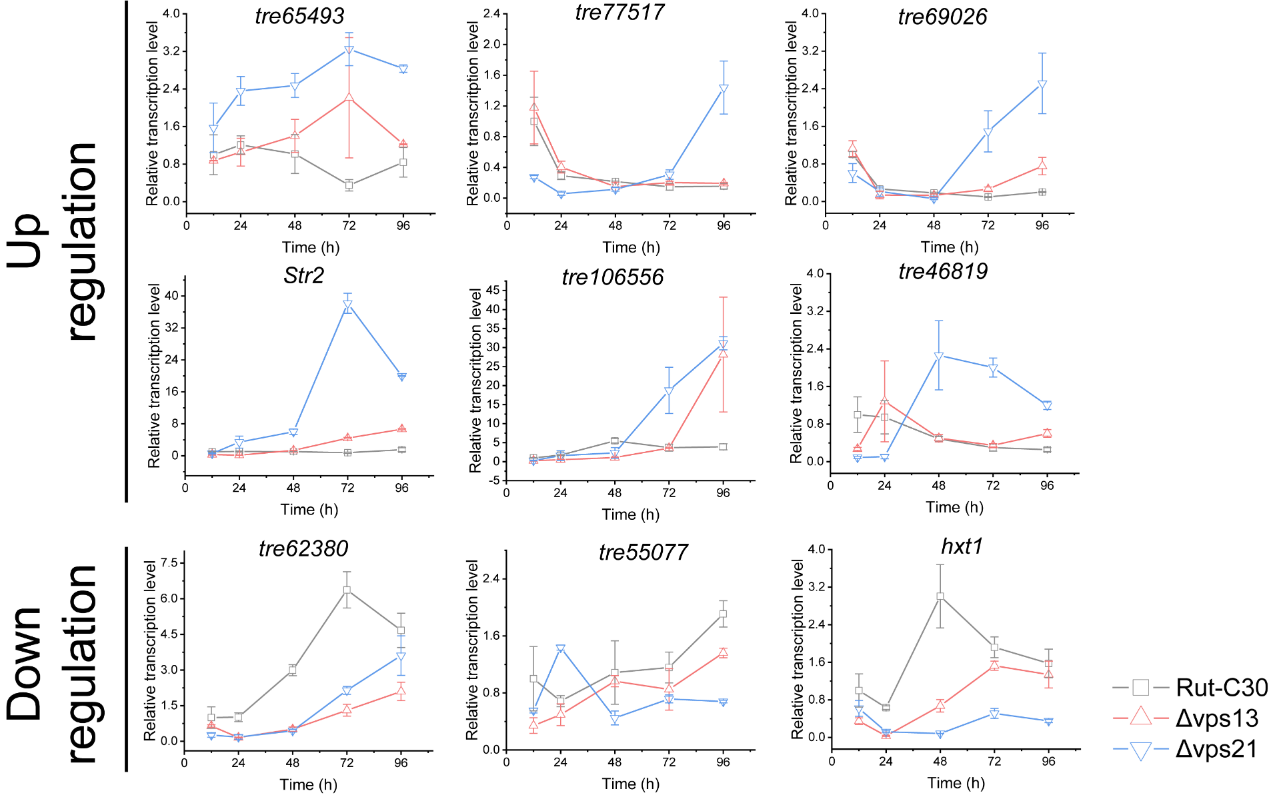


**Fig. S1 Transcription level of sugar transporters during cellulase induction on Avicel inducing medium.** The parent strain Rut-C30 and its *vps13* or *vps21* deleting mutants (Δ*vps13* or Δ*vps21*) were precultured in SDB broth for mycelia accumulation, equal amounts of mycelia were transferred to Avicel inducing medium, samples were taken at 12 h, 24 h, 48 h, 72 h, and 96 h for transcription level analysis. The transcription level of each transporter gene in Rut-C30 at 12 h was normalized as 1. Cellulase genes were upregulated in Δ*vps13* and Δ*vps21* mutants, and nine sugar transporters were differentially expressed in late inducing stage (after 72 h). Among that, the expression of *tre65493*, *tre77517*, *tre69026*, *str2*, *tre106556* and *tre46819* was upregulated especially in late inducing stage in Δ*vps13* or Δ*vps21*, and the transporters gene *tre62380*, *tre55077* and *hxt1* were downregulated during the cellulase induction. Values are presented as the mean with the standard deviation from three biological replicates.


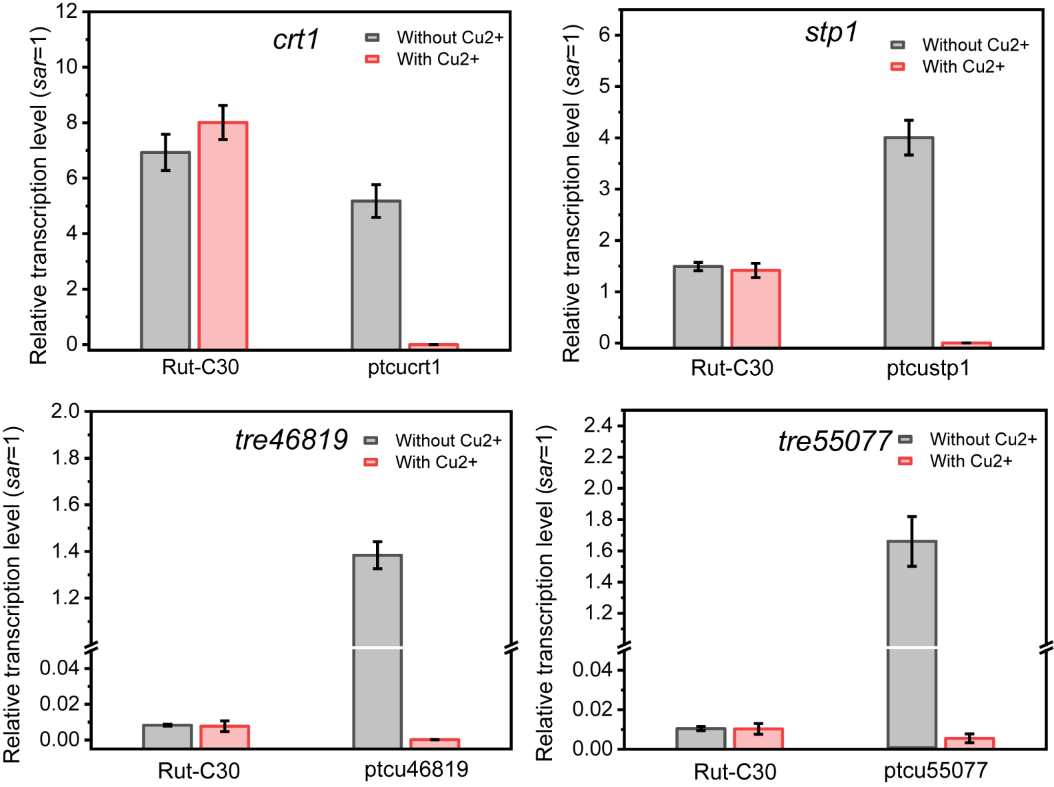


**Fig. S2 Analysis of the expression of Ptcu controlled transporters.** The parent strains and four randomly selected transformants were inoculated in the Avicel inducing medium for 36 h before RNA isolation. 20 μM CuSO_4_ was added to repress the expression of transporter. The transcription level of endogenous *sar1* was set as 1. Three replicates were applied for each reaction. Values are presented as the mean.


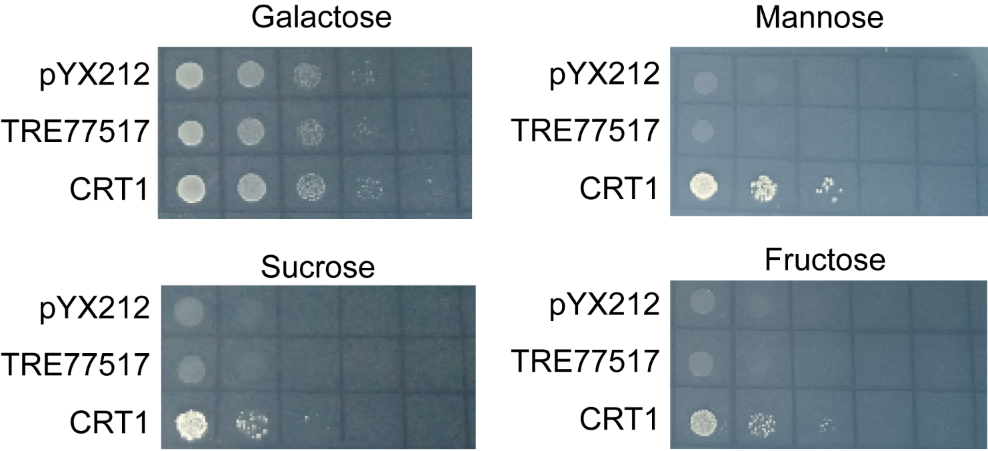


**Fig. S3 Sugar transport analysis of TRE77517 for other mono/disaccharides.** S. cerevisiae strains were precultured in SC medium with 20 g/L maltose as carbon source for 16 h. cells were harvest and washed twice, diluted to the initial OD_600_=0.5. 10-fold serial dilution was applied, and 5 μL suspension was dropped on the SC plate with 20 g/L galactose, mannose, fructose, and sucrose as carbon source.


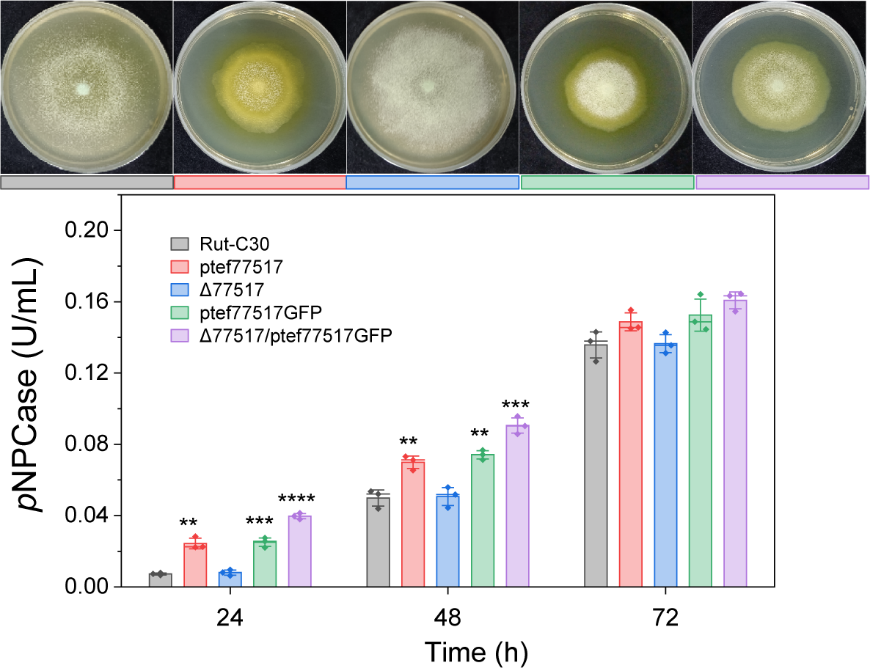


**Fig. S4 Plate assay and cellulase induction by overexpressing TRE77517GFP in *T. reesei*.** Upper panel, plate assay for strains Rut-C30, ptef77517, Δ77517, ptef77517GFP and Δ77517/ptef77517GFP. Approximate 10^7^ spores were inoculated in the PDA plate and cultured in 30 °C for 96 h. Lower panel, *p*NPCase activity during cultured in minimal medium with 1% Avicel as sole carbon source. Three biological replicates were applied for each strain, and the data were represented as mean ± S.D. ***p* < 0.01, ****p* < 0.001, *****p* < 0.0001, Student’s t test.


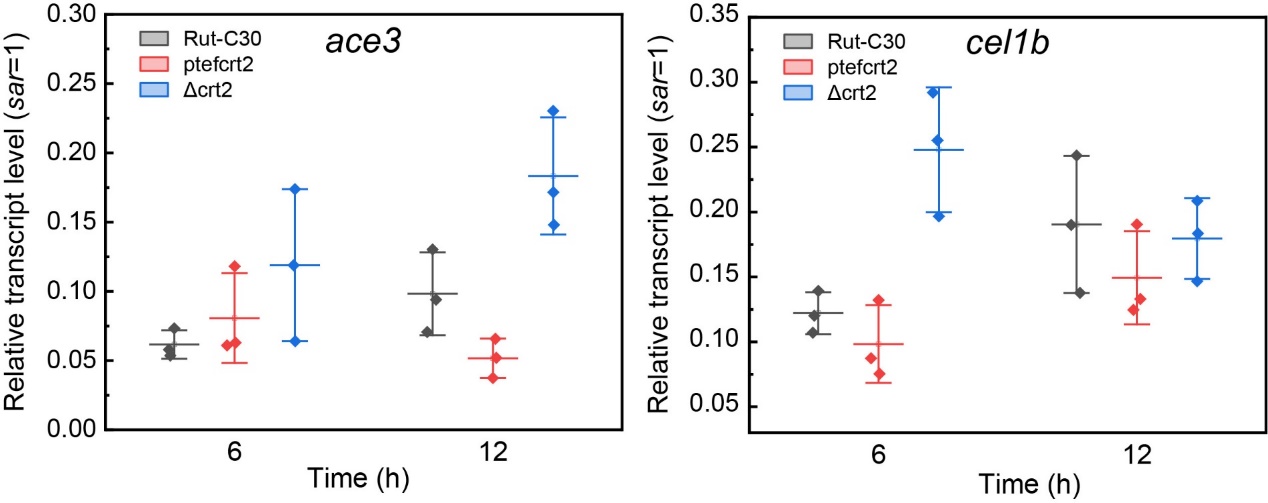


**Fig. S5 Transcription of the two adjacent gene *ace3* and *cel1b* during cellulase induction.** Strain Rut-C30, ptefcrt2 and Δcrt2 are precultured in MM medium with glucose as carbon sources, and then transferred to a fresh MM medium with Avicel as carbon source. Samples were taken at indicated time and subjected to RT-qPCR analysis. The expression of *sar1* was kept as 1. Results were represented as mean value from three individual biological replicates.


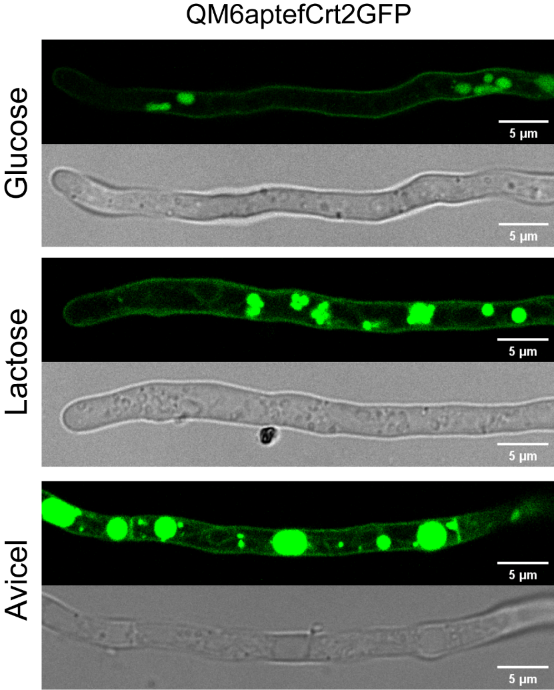


**Fig. S6 Subcellular location of CRT2GFP (TRE77517GFP) in QM6a.** C-terminal GFP fused Crt2 was expressed under the control of strong constitutive promoter Ptef in QM6a, resulting in strain QM6aptefcrt2GFP. QM6aptefcrt2GFP was cultured in MM medium plus 2 g/L tryptone for 18 h when glucose and lactose was used as carbon source, and for 24 h in Avicel medium. Images were taken under a 63×oil objective. Scale bar represents 5 μm.





**Fig. S7** **Dysfunction of CRT2 (TRE77517) affect cellulase induction in the wild type QM6a**. QM6a, QM6aptefcrt2, and QM6aΔcrt2 were pre-cultured in MM medium with 10 g/L glucose and 2 g/L tryptone for mycelia accumulation for 48 h. Equal amounts of mycelia were transferred to MM medium containing 10 g/L Avicel, samples were taken after 6 h induction and subjected to RT-qPCR analysis. The expression of *sar1* was kept as 1. Results were represented as mean value from three individual biological replicates. Data were analyzed using t-test, n.s. not significant (p > 0.05), **p* < 0.05, ***p* < 0.01, Student’s t test.





**Fig. S8 The expression of *crt2* (*tre77517*) was activated in cellulase-induced carbon sources.** Parent strain Rut-C30 were precultured in MM medium with glucose as carbon sources for 48 h, and equal amounts of mycelia were transferred to MM medium with 10 g/L glucose, cellobiose, lactose and Avicel as sole carbon source. Samples were taken at 6 h and 12 h and analyzed for transcription level. The transcription of *sar1* was set as 1. Results were represented as mean value from three individual biological replicates.

**Table S1 Strains and plasmids used in this study**

| **Strains** | **Genotype** |
| --- | --- |
| Rut-C30 | Hyper cellulase producer of *Trichoderma reesei* |
| C30Δura3 | Rut-C30, *ura3^—^* |
| ptcucrt1 | Rut-C30, *ura3^—^*, *Ptcu-crt1*::*URA3* |
| ptcustp1 | Rut-C30, *ura3^—^*, *Ptcu-stp1*::*URA3* |
| ptcustr2 | Rut-C30, *Ptcu-str2*::*Hyg^r^* |
| ptcu46819 | Rut-C30, *ura3^—^*, *Ptcu-tre46819*::*URA3* |
| ptcu55077 | Rut-C30, *ura3^—^*, *Ptcu-tre55077*::*URA3* |
| ptcu62380 | Rut-C30, *ura3^—^*, *Ptcu-tre62380*::*URA3* |
| ptcu65493 | Rut-C30, *Ptcu-tre65493*::*Hyg^r^* |
| ptcu69026 | Rut-C30, *ura3^—^*, *Ptcu-tre69026*::*URA3* |
| ptcu77517 | Rut-C30, *ura3^—^*, *Ptcu-tre77517/crt2*::*URA3* |
| ptcu106556 | Rut-C30, *ura3^—^*, *Ptcu-tre106556*::*URA3* |
| ptef77517a | Rut-C30, *ura3^—^*, *Ptef-tre77517*(*crt2*)*-Tcbh1*::*URA3* |
| ptef77517b(ptefcrt2) | Rut-C30, *ura3^—^*, *Ptef-tre77517*(*crt2*)*-Tcbh1*::*URA3* |
| Δ77517(Δcrt2) | Rut-C30, *ura3^—^*, Δ*tre77517/crt2*::*URA3* |
| Δcrt1 | Rut-C30, Δ*crt1*::*Hyg^r^* |
| Δxyr1 | Rut-C30, Δ*xyr1*::*Hyg^r^* |
| ptefcrt2Δcrt1 | Rut-C30, *ura3^—^*, *Ptef-crt2-Tcbh1*::*URA3*; Δ*crt1*::*Hyg^r^* |
| ptefcrt2Δxyr1 | Rut-C30, *ura3^—^*, *Ptef-crt2-Tcbh1*::*URA3*; Δ*xyr1*::*Hyg^r^* |
| ptef77517GFP | Rut-C30, *Ptef-crt2-gfp-Tcbh1*::*Hyg^r^* |
| Δ77517/ptef77517GFP | Rut-C30, *ura3^—^*, Δ*crt2*::*URA3*; *Ptef-crt2-gfp-Tcbh1*::*Hyg^r^* |
| OExyr1 | Rut-C30, *ura3^—^*, *PgpdA*-*xyr1*::*URA3* |
| QM6a | Wild type *T. reesei* |
| QM6aptefcrt2 | QM6a, *Ptef-crt2-Tcbh1*::*Hyg^r^* |
| QM6aΔcrt2 | QM6a, Δ*crt2*::*Hyg^r^* |
| QM6aΔcre1 | QM6a, Δ*cre1*::*Hyg^r^* |
| QM6aptefcrt2GFP | QM6a, *Ptef-crt2-gfp-Tcbh1*::*Hyg^r^* |
| EBY.VW4000 | CEN.PK2-1C (*MATα leu2-3, 112 ura3-52 trp1-289 his3-Δ1 MAL2-8^c^ SUC2 hxt17Δ*) *hxt13Δ::loxP hxt15Δ::loxP hxt16Δ::loxP hxt14Δ::loxP hxt12Δ::loxP hxt9Δ::loxP hxt11Δ::loxP hxt10Δ::loxP hxt8Δ::loxP hxt514Δ::loxP hxt2Δ::loxP hxt367Δ::loxP gal2 Δ stl1Δ::loxP agt1Δ::loxP ydl247wΔ::loxP yjr160cΔ::loxP* |
| EBY/gh1-1 | EBY.VW4000, *Ptpi-gh1-1*::*HIS3* |
| EBY/gh1-1/pYX212 | EBY.VW4000, *Ptpi-gh1-1*::*HIS3*; pYX212::*URA3* |
| EBY/gh1-1/Crt1 | EBY.VW4000, *Ptpi*-*gh1-1*::*HIS3*; pYX212-*Ptpi-crt1*::*URA3* |
| EBY/gh1-1/Tre77517 | EBY.VW4000, *P_TPI_*-*gh1-1*::*HIS3*; pYX212-*Ptpi-tre77517*::*URA3* |
| EBY/gh1-1/Tre77517GFP | EBY.VW4000, *Ptpi*-*gh1-1*::*HIS3*; pYX212-*Ptpi-tre77517-gfp*::*URA3* |
| **Plasmid** | **Cassette** |
| pCAMBIA1301G_Ptcucrt1 | *ΔPcrt1::Ptcu-crt1, URA3, Kan^r^* |
| pCAMBIA1301G_Ptcustp1 | *ΔPstp1::Ptcu-stp1, URA3, Kan^r^* |
| pCAMBIA1301G_Ptcustr2 | *ΔPstr2::Ptcu-str2, Hyg^r^, Kan^r^* |
| pCAMBIA1301G_Ptcu46819 | *ΔPtre46819::Ptcu tre46819, URA3, Kan^r^* |
| pCAMBIA1301G_Ptcu55077 | *ΔPtre55077::Ptcu-tre55077, URA3, Kan^r^* |
| pCAMBIA1301G_Ptcu62380 | *ΔPtre62380::Ptcu-tre62380, URA3, Kan^r^* |
| pCAMBIA1301G_Ptcu65493 | *ΔPtre65493::Ptcu-tre65493, Hyg^r^, Kan^r^* |
| pCAMBIA1301G_Ptcu69026 | *ΔPtre69026::Ptcu-tre69026, URA3, Kan^r^* |
| pCAMBIA1301G_Ptcu77517 | *ΔPtre77517::Ptcu-tre77517, URA3, Kan^r^* |
| pCAMBIA1301G_Ptcu106556 | *ΔPtre106556::Ptcu-tre106556, URA3, Kan^r^* |
| pCAMBIA1301G_Ptef77517 | *Ptef-tre77517-Tcbh1, URA3, Kan^r^* |
| pCAMBIA1301G_Δ77517 | *Δtre77517::URA3, Kan^r^* |
| pCAMBIA1301G_Δcrt1 | *Δcrt1::Hyg^r^, Kan^r^* |
| pCAMBIA1301G_Δxyr1 | *Δxyr1::Hyg^r^, Kan^r^* |
| pCAMBIA1301G_Δcre1 | *Δcre1::Hyg^r^, Kan^r^* |
| pCAMBIA1301G_Ptef77517_hyg | *Ptef-tre77517-Tcbh1, Hyg^r^, Kan^r^* |
| pCAMBIA1301G_Δ77517_hyg | *Δtre77517::Hyg^r^, Kan^r^* |
| pCAMBIA1301G_Ptef77517GFP | *Ptef-tre77517-GFP-Tcbh1, Hyg^r^, Kan^r^* |
| pYX212-Tre77517 | *Ptpi-tre77517, URA3, Amp^r^, 2μ* |
| pYX212-Tre77517GFP | *Ptpi-tre77517-GFP, URA3, Amp^r^, 2μ* |
| pYX212-Crt1 | *Ptpi-crt1, URA3, Amp^r^, 2μ* |

**Table S2 Primers used in this study**

| **Primers** | **Sequences (5’-3’)** | **Target** |
| --- | --- | --- |
| Ura3_F | aattcgcgttaacggtgagactagcggccg | URA3 selection marker |
| Ura3_R | tcgtacctggtttccaggtgctcctgg |  |
| Ptcu_F | gcacctggaaaccaggtacgatgccgattcgtgaatcctg | Ptcu promoter |
| Ptcu_R | gagctctgtcgtatcaaccaggtcgtataga |  |
| Hyg_F | gatctggattttagtactggattttggtt | Ptcu promoter and Hyg^r^ selection marker |
| Ptcu_R2 | gagctctgtcgtatcaaccaggtcgtatagat |  |
| Pcrt1_up_F | attcgggggaattcgcgtctatgtcgacccggtccatcc | Upstream flank of Pcrt1 promoter |
| Pcrt1_up_R | ctagtctcaccgttaacgcgaattttgccctggttgcatgtcag |  |
| Pcrt1_down_F | acctggttgatacgacagagctcatgggcgagaaagaagacattcac | Downstream flank of Pcrt1 promoter |
| Pcrt1_down_R | acaggattcaatcttaatacgtacctgatcccaccagcgcttatcgg |  |
| Pstp1_up_F | attcgggggaattcgcgtggctccacctgtacatattggcat | Upstream flank of Pstp1 promoter |
| Pstp1_up_R | ctagtctcaccgttaacgcgaattgagattgaggtgcgcctgtgc |  |
| Pstp1_down_F | acctggttgatacgacagagctcatggccgacgtccacgtcgc | Downstream flank of Pstp1 promoter |
| Pstp1_down_R | caggattcaatcttaatacgtacctggagcagacgttgaccagggaga |  |
| P46819_up_F | ttcgggggaattcgcgtgacgcacgtgggatccaacaa | Upstream flank of P46819 promoter |
| P46819_up_R | agtctcaccgttaacgcgaattccaagccaagcaacatctgtga |  |
| P46819_down_F | cctggttgatacgacagagctcatgggaatcctcaaacccacca | Downstream flank of P46819 promoter |
| P46819_down_R | acaggattcaatcttaatacgtacctggcggttgacgagcagggatac |  |
| P55077_up_F | ttcgggggaattcgcgtgttgaggctgagaaacacccccc | Upstream flank of P55077 promoter |
| P55077_up_R | agtctcaccgttaacgcgaatttgagatggcgctggatgcatg |  |
| P55077_down_F | cctggttgatacgacagagctcatggaaaagtccactctggccg | Downstream flank of P55077 promoter |
| P55077_down_R | acaggattcaatcttaatacgtacctggccaaagtacggcatgacgaag |  |
| P62380_up_F | ttcgggggaattcgcgtaggaatgtggtttggtggtgga | Upstream flank of P62380 promoter |
| P62380_up_R | caaaatccagtactaaaatccagatccaagggtactttgtctgccggt |  |
| P62380_down_F | acctggttgatacgacagagctcatgcgttccagaaggggcg | Downstream flank of P62380 promoter |
| P62380_down_R | acaggattcaatcttaatacgtacctgttatcagacgatcgaggcgcca |  |
| P65493_up_F | ttcgggggaattcgcgtcaaatcagtgacattcgtccccc | Upstream flank of P65493 promoter |
| P65493_up_R | ccaaaatccagtactaaaatccagatcaggtgccacagtggtacatgt |  |
| P65493_down_F | tggttgatacgacagagctcatggagacgacgattcctccc | Downstream flank of P65493 promoter |
| P65493_down_R | acaggattcaatcttaatacgtacctgagggctgggacgtggataagc |  |
| P69026_up_F | ttcgggggaattcgcgtgtcgacagatgagaacgacgagg | Upstream flank of P69026 promoter |
| P69026_up_R | gtctcaccgttaacgcgaattgactgtgcatcgagcgtgtgat |  |
| P69026_down_F | cctggttgatacgacagagctcatgcgcttcttcaagaactatcgc | Downstream flank of P69026 promoter |
| P69026_down_R | acaggattcaatcttaatacgtacctgatgcccaggatcaggaacaggc |  |
| P77517_up_F | ttcgggggaattcgcgtaaagtccctgctgtagacggc | Upstream flank of P77517 promoter |
| P77517_up_R | tctcaccgttaacgcgaattctcgacggagtaaatgggctcc |  |
| P77517_down_F | tggttgatacgacagagctcatggctccctctgcggcgc | Downstream flank of P77517 promoter |
| P77517_down_R | acaggattcaatcttaatacgtacctgagcgaaacacctccagagcc |  |
| P106556_up_F | ttcgggggaattcgcgtatctcacgtccgcttgacgtc | Upstream flank of P106556 promoter |
| P106556_up_R | tctcaccgttaacgcgaattttttcctgctcggcccacgat |  |
| P106556_down_F | tggttgatacgacagagctcatgggattcttgaacaaaaaagccga | Downstream flank of P106556 promoter |
| P106556_down_R | acaggattcaatcttaatacgtacctgagaccgatgatggtggcaaaca |  |
| Pstr2_up_F | ttcgggggaattcgcgtaagacgttggttacgccctcg | Upstream flank of Pstr2 promoter |
| Pstr2_up_R | caaaatccagtactaaaatccagatctgccctatgacaacaactggagt |  |
| Pstr2_down_F | tggttgatacgacagagctcatgtcgtcaaatccagcaggcg | Downstream flank of Pstr2 promoter |
| Pstr2_down_R | caggattcaatcttaatacgtacctgcaagcacgtgggaaacactcttg |  |
| Ptef_F | ttcgggggaattcgcgtacatcaccagcgtcaggacac | Ptef1 promoter |
| Ptef_F | tttgacggtttgtgtgatgtagcg |  |
| Crt2_F | acatcacacaaaccgtcaaaatggctccctctgcggcgc | Crt2 CDS |
| Crt2_R | ttcgccacggagctgagctcttatacagatgaaccggaaaccgact |  |
| Delcrt2_up_F | ttcgggggaattcgcgttgacagagtttgctggaggtgc | Upstream flank of crt2 |
| Delcrt2_up_R | ccaggagcacctggaaacctcttccaggagctgcgacag |  |
| Delcrt2_down_F | ccgctagtctcaccgttattatagacggggcatggcgtt | Downstream flank of crt2 |
| Delcrt2_down_R | acaggattcaatcttaatacgtacctggtgaacttgctccgaaagttgc |  |
| Delcrt1_up_F | ttcgggggaattcgcgtatacctgccattagccgaaccg | Upstream flank of Crt1 |
| Delcrt1_up_R | tccagatcgagctctacgaggcagcgttgaaggaaagcaa |  |
| Delcrt1_down_F | accaacggatcggtcggtatctgggcagttgtgcgcag | Downstream flank of Crt1 |
| Delcrt1_down_R | caggattcaatcttaatacgtacctgctgagaagctgggggagagct |  |
| Delxyr1_up_F | ttcgggggaattcgcgtgatcgtgatgtggcagccgg | Upstream flank of Xyr1 |
| Delxyr1_up_R | atccagatcgagctctacgtgtgaagctgttgaacgtatggt |  |
| Delxyr1_down_F | accaacggatcggtcggtcgtatgacgttggattggggga | Downstream flank of Xyr1 |
| Delxyr1_down_R | caggattcaatcttaatacgtacctgtgggggggaaagcaaaaggtaag |  |
| Delcre1_up_F | attcgggggaattcgcgtgaccgtggtttccaccgtgg | Upstream flank of Cre1 |
| Delcre1_up_R | atccagatcgagctctacgagaagcgagtgtgggtttttgg |  |
| Delcre1_down_F | accaacggatcggtcggtggcgcgcatcgaatgacttac | Downstream flank of Cre1 |
| Delcre1_down_R | caggattcaatcttaatacgtacctgaaaagtgcgcgattgcgtgta |  |
| pYXCrt1_F | ctacaaaaaacacatacaggaattcatgggcgagaaagaagacattcac | Crt1 CDS |
| pYXCrt1_R | cttgtgggccctaggatccttaagccttctcgatattgacaatgtcg |  |
| pYX77_F | actacaaaaaacacatacaggaattcatggctccatctgctgc | Synthesized tre77517 CDS |
| pYX77gfp_R | gcccttgctcacggatccacctccgccaccagaaacggaagaaccggaaacgg |  |
| Gfp_F | ggatccgtgagcaagggcga | Gfp CDS |
| Gfp_R | gcttgtgggccctaggatccttacttgtacagctcgtccatgcc |  |
| **Primers for qPCR** | **Sequences (5’-3’)** | **Target** |
| qsar1_F | tggatcgtcaactggttctacga | *sar1* |
| qsar1_R | tgtgtagcaacgtggtcttt |  |
| qcel7a_F | gcggatcctctttctcagac | *cel7a* |
| qcel7a_R | ttggcgtagtaatcatccca |  |
| qcrt2_F | attgacgggtcgatgacca | *crt2(tre77517)* |
| qcrt2_R | gcatctgtcgctgatccagc |  |
| qcrt1_F | cgcgttggcgccatgtacaa | *crt1* |
| qcrt1_R | gatgatgatgaggccggagaa |  |
| qxyr1_F | taccaagtgcgatggcttac | *xyr1* |
| qxyr1_F | ctctctcggacatattcgca |  |
| qcre1_F | tggctgcagcaagaagttct | *cre1* |
| qcre1_R | gggtgcatcaagccatcga |  |
